# Mechanistic Insights into the Photocatalytic Indigo Carmine Dye Decolorization by Co_3_O_4_/TiO_2_

**DOI:** 10.1002/cphc.202400688

**Published:** 2025-03-03

**Authors:** Mirjam E. de Graaf, Nejc Godec, Bram T. Kappé, Roos L. Grote, Jitte Flapper, Eline M. Hutter, Bert M. Weckhuysen

**Affiliations:** ^1^ Inorganic Chemistry and Catalysis group,< Institute for Sustainable and Circular Chemistry Utrecht University Universiteitsweg 99 3584 CG Utrecht, The Netherlands; ^2^ AkzoNobel Decorative Coatings B.V. Rijksstraatweg 31 2171 AJ Sassenheim, The Netherlands

**Keywords:** Photocatalysis, Dye decolorization, Metal oxide, Cobalt, Titania

## Abstract

TiO_2_ is widely studied as an efficient UV‐light photocatalyst for organic compound degradation through reactive oxygen species (ROS) generation. TiO_2_ can be modified to show photocatalytic activity under visible light illumination by combining with visible‐light absorbing metal oxides. Here, we investigated Co_3_O_4_/TiO_2_ composite materials as visible‐light absorbing photocatalysts, with various weight loadings of Co_3_O_4_, for the decolorization of wastewater pollutant indigo carmine. Under green LED light, 1.4 wt% Co_3_O_4_/TiO_2_ showed the highest decolorization rate compared to other weight loadings and bare TiO_2_. While UV‐Vis spectroscopy indicated that Co_3_O_4_/TiO_2_ composite materials and bare TiO_2_ cause similar dye decolorization behavior, NMR spectroscopy showed that after 24 h, reaction products were present in the reaction mixture for 1.4 wt% Co_3_O_4_/TiO_2_, while TiO_2_ showed no reaction products. The lack of photocatalytic activity of Co_3_O_4_/zeolite and other Co_3_O_4_/oxide composite materials suggests a synergistic effect between Co_3_O_4_ and TiO_2_, where a small amount of Co_3_O_4_ enables TiO_2_ to utilize visible light without compromising the surface area available for ROS creation. Lastly, we emphasize the need to be cautious when drawing conclusions regarding the dye degradation, since we showed that decolorization does not necessarily equate to full degradation, using a unique combination of UV‐Vis and nuclear magnetic resonance spectroscopy.

## Graphical abstract

Visible‐light photocatalysts eliminate the need for additional UV‐lighting in photocatalysis. Both 1.4 wt% Co_3_O_4_/TiO_2_ and TiO_2_ catalysts fully decolorize a 100 ppm aqueous solution of indigo carmine dye under green light illumination within several hours, but proton nuclear magnetic resonance (NMR) combined with UV‐Vis suggest that the degradation mechanism is different for each catalyst.

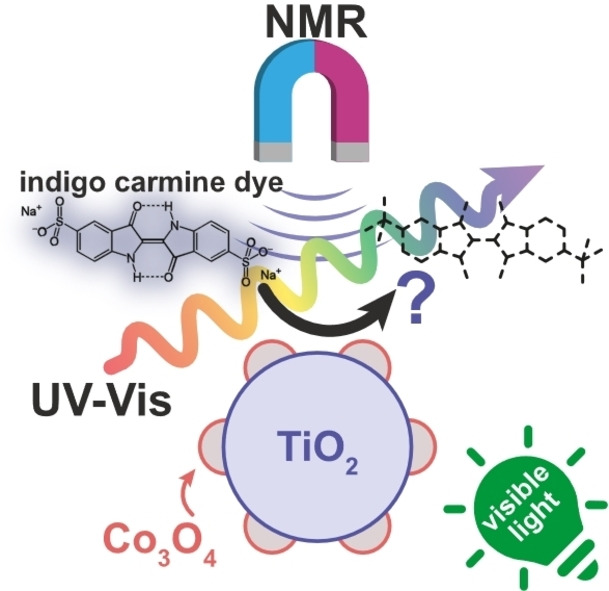

## Introduction

TiO_2_ is used as a photocatalytic standard in industry because of its high photocatalytic activity under UV light.[^1^, ^2^] However, TiO_2_ typically shows little to no activity under indoor lighting conditions, since standard indoor light sources (*e. g*., LED lamps) only emit visible light. Therefore, designing photocatalysts that utilize the spectrum of indoor light sources will eliminate the need for additional UV‐lighting. TiO_2_ can be modified to have visible‐light absorbing properties, for example by decorating the surface with graphitic carbon nitrate, by doping with (transition) metals (*e. g*., Fe, Mn, Co, Cr) or with non‐metals (*e. g*., N, C, P), or by combining with other, visible‐light absorbing metal oxides (*e. g*., Co_3_O_4_, Fe_2_O_3_, MnO_2_).[^3^, ^4^, ^5^, ^6^, ^7^, ^8^, ^9^, ^10^, ^11^]

In previous literature, the removal of dye molecules from (waste)water has been studied frequently.[^12^, ^13^, ^14^, ^15^, ^16^, ^17^] Specifically, Co_3_O_4_/TiO_2_ materials have been shown to be active for dye removal, as well as for, *e. g*., NO_x_ reduction, water splitting, and degradation of antibiotics.[^18^, ^19^, ^20^, ^21^] Studies investigating dye removal generally focus only on the decolorization of the dye by light absorption spectroscopy, whereas dye molecules can be decolorized without full chemical decomposition.^[22]^ Only a limited number of publications report on dye decolorization over various catalysts combined with reaction product analysis methods like high‐performance liquid chromatography or gas chromatography‐mass spectrometry.[^23^, ^24^, ^25^] Here, we study the photocatalytic decolorization of indigo carmine dye over a Co_3_O_4_/TiO_2_ photocatalyst by combining dye decolorization analysis by UV‐Vis spectroscopy with chemical decomposition analysis of the dye molecules using nuclear magnetic resonance (NMR) spectroscopy. NMR spectroscopy may provide valuable information on reaction products and/or intermediates, as well as information the degradation mechanism through analyzing the proton‐proton coupling. The combination of both techniques is unique for dye decolorization reactions over Co_3_O_4_/TiO_2_ photocatalysts. Table S1 presents an overview of various composite photocatalyst materials used for indoor pollutant removal, as cited in this study, emphasizing the innovative aspects of our research work.

Metal oxides such as iron oxide, manganese oxide and cobalt oxide are among the few metal oxides that naturally absorb visible light.[^26^, ^27^, ^28^, ^29^] Here, we investigated whether composites of cobalt(II,III) oxide (Co_3_O_4_) and TiO_2_ (Co_3_O_4_/TiO_2_) are suitable catalysts for the photocatalytic oxidation of organic compounds under visible light illumination. Additionally, similar composites of Co_3_O_4_ and five other materials than TiO_2_ (zeolites ZSM‐5, Y and Beta, and oxides SiO_2_ and Al_2_O_3_) were screened for their photocatalytic activity. Composite materials of iron oxide and manganese oxide with TiO_2_ (M_x_O_y_/TiO_2_, where M=Fe, Mn) were also prepared, but we observed no photocatalytic activity for these composite materials (Figure S2).

As determined by the International Organization for Standardization (ISO), methylene blue decolorization is a test system to determine the photocatalytic activity of surfaces in aqueous media.[^30^, ^31^] Dye molecules are typically decolorized through oxidation reactions with reactive oxygen species, which are formed at the catalyst surface.[^10^, ^32^, ^33^] We tested the photocatalyst composite materials for their photocatalytic activity by decolorizing indigo carmine dye as a model reaction. Indigo carmine is a commonly used dye in the textile industry and thus often ends up in wastewater streams.[^34^, ^35^, ^36^] Since indigo carmine can have several adverse effects on human health and the environment, it is important that it is removed from wastewater completely without leaving behind (more) harmful byproducts.

Although many studies have been conducted on dye decolorization reactions on TiO_2_[^37^, ^38^, ^39^, ^40^, ^41^], the effect of the controlled addition of Co_3_O_4_ is not known, even though the ratio between Co_3_O_4_ and TiO_2_ can have a significant impact on the observed rate of dye decolorization.[^13^, ^42^, ^43^, ^44^] Therefore, we performed an indigo carmine dye decolorization reaction using Co_3_O_4_/TiO_2_ composite materials with different weight percentages of Co_3_O_4_ (*i. e*., *x* wt % Co_3_O_4_/TiO_2_, where 0.14 < *x* <14). To this end, the photocatalyst powders were mixed with an aqueous solution of indigo carmine, after which the mixture was illuminated with simulated solar light or green LED light.

In this work, we varied the weight percentage of Co_3_O_4_ to find the optimal Co_3_O_4_/TiO_2_ ratio, *i. e*., the material that shows the highest rate of dye decolorization. Furthermore, we shed light on the decolorization mechanism, as well as the differences between the decolorization process using a 1.4 wt% Co_3_O_4_/TiO_2_ catalyst *vs*. a bare TiO_2_ catalyst. To this end, we compared UV‐Vis spectra to proton nuclear magnetic resonance (^1^H‐NMR) spectra, and we related dye decolorization in the form of decreasing UV‐Vis absorbance to dye degradation, *i. e*., the complete photo‐oxidation of the organic dye into CO_2_, using ^1^H‐NMR.

## Results and Discussion

**Figure 1:**
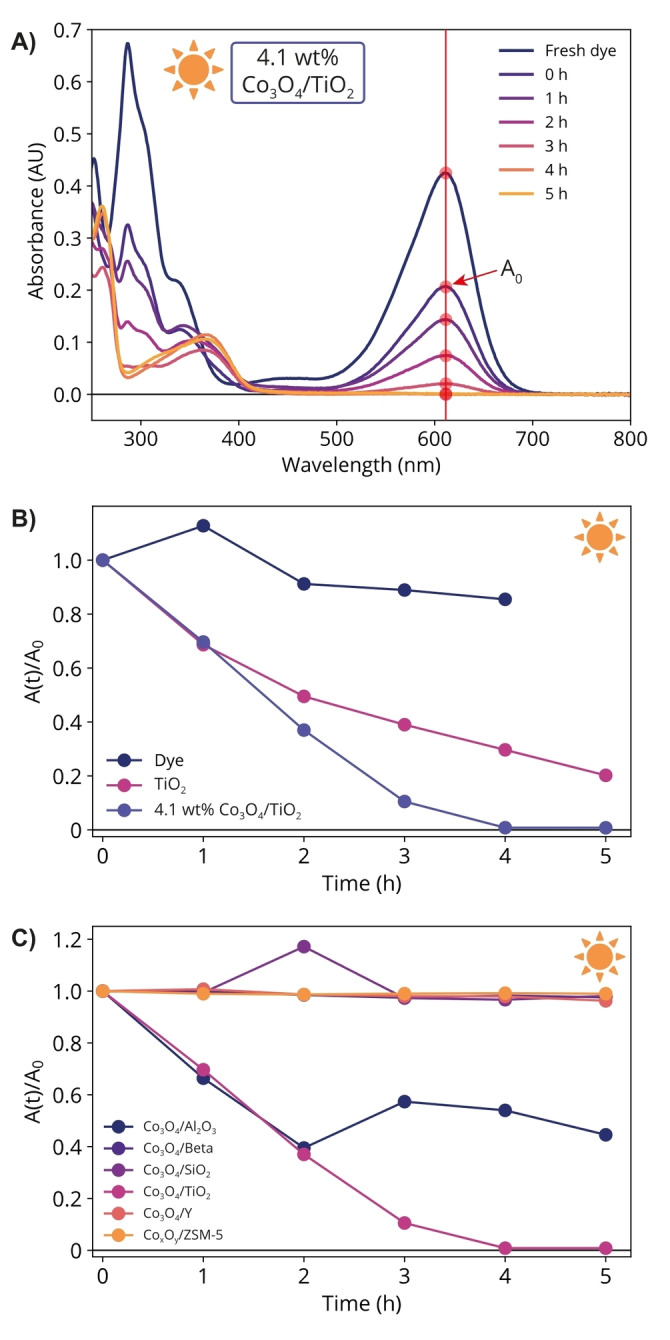
A) UV‐Vis absorption spectra of an indigo carmine dye decolorization reaction, illustrating the data processing procedure used for all dye decolorization reactions in this work. 100 ppm indigo carmine dye was dissolved in water, mixed with 10 mg catalyst per mL dye, equilibrated in the dark overnight, and then illuminated with simulated solar light or green light. For the reaction shown in this figure, a 4.1 wt % Co_3_O_4_/TiO_2_ catalyst was used under simulated solar light illumination. Absorption spectra are shown for the fresh dye, which has not been in contact with the catalyst, and for all aliquots taken every hour. The height of the absorption band at 611 nm (A(t)) was determined for each sample as indicated by the red line and circles. The degree of decolorization can be determined by normalizing the absorbance at a time t >0 h (A(t)) to the absorbance at t = 0 h (A_0_). B) Dye decolorization performed using a 4.1 wt% Co_3_O_4_/TiO_2_ composite and bare TiO_2_. A(t)/A_0_ is also shown for the dye without any catalyst. C) Dye decolorization performed using composite materials of Co_3_O_4_ with different zeolite and oxide materials. For the composite material containing ZSM‐5, the cobalt oxidation state was not confirmed with diffuse reflectance spectroscopy. Therefore, cobalt oxide is referred to as Co_x_O_y_ in this material.

Figure 1 shows the UV‐Vis absorption spectra of a dye decolorization reaction (catalyst: 4.1 wt % Co_3_O_4_/TiO_2_, light source: solar simulator, AM1.5G, 1 Sun intensity) to illustrate how the absorption spectra were processed throughout this work. In general, a 100 ppm solution of indigo carmine dye in water was mixed with 10 mg catalyst per mL dye solution and left to equilibrate overnight in the dark, after which illumination was started. After the powdered catalyst was removed from the aliquot through centrifugation, absorption spectra were recorded, once directly before illumination (0 h) and then for every aliquot taken. To determine the degree of dye decolorization, the absorbance value A(t) (red circles in Figure 1A) was followed over time at a fixed wavelength of 611 nm, and A(t) is normalized to the absorbance at t = 0 (A_0_). During the equilibration process in the dark, some dye molecules adsorb at the catalyst surface, resulting in an absorbance decrease (fresh vs. 0 h). After equilibration, the absorbance did not change over time under dark conditions (Figure S4). Once illumination is started, the absorbance decrease is therefore attributed to decolorization of the dye. There is no physical reason for the absorbance to increase during the photocatalytic degradation process. We therefore attribute any observed increase in absorbance (A(t)/A_0_ >1) to measurement error or to concentration fluctuations in the reaction mixture.

Common practice in literature is to express the degree of decolorization in terms of concentration (C/C_0_),[^12^, ^45^, ^46^, ^47^] since light absorption relates directly and linearly to concentration through the Beer‐Lambert law. However, we deliberately express the degree of decolorization in terms of absorbance values (A(t)/A_0_), as dye decolorization does not necessarily equate to dye degradation, *i. e*., chemical decomposition of the molecule. For example, methyl viologen dye can be decolorized due to an electron residing on the molecule without being chemically degraded.[^22^, ^48^]

Figure 1B shows the indigo carmine dye absorbance A(t) as a function of illumination time (simulated sunlight, AM1.5G) normalized to the initial absorbance A_0_. Without photocatalyst (dye only), we observed a ~10 % reduction in dye absorbance in the first 4 h, which we attribute to self‐degradation of the dye (Figure S6). We found that bare TiO_2_ partially decolorizes the dye molecule under solar light illumination within 5 h. While TiO_2_ does not achieve full dye decolorization within the measured timeframe, from Figure 1B it was estimated that full decolorization takes place within ~10 h. This is comparable to literature, where TiO_2_ typically decolorizes dyes in several minutes to several hours, depending on *e. g*. the amount of catalyst used per mL dye, the initial dye concentration and the light source used.[^39^, ^49^, ^50^, ^51^, ^52^] It should be noted that our solar simulator contains only a few percent of UV‐light (λ < 400 nm), which is (partially) absorbed by TiO_2_, and that a UV light source of the same intensity as our solar simulator would enable faster decolorization using TiO_2_. Interestingly, the 4.1 wt % Co_3_O_4_/TiO_2_ material decolorized the dye completely in ~4 h when using solar light.

To separate the contributions of Co_3_O_4_ and TiO_2_ to the decolorization of indigo carmine, we tested composite materials of cobalt oxide with common support materials for their ability to decolorize indigo carmine dye as a model dye to evaluate photocatalytic activity. To this end, composite materials of cobalt oxide and six other materials, including TiO_2_, were synthesized in a wet impregnation method. The materials used were zeolite ZSM‐5, zeolite Y, zeolite Beta, SiO_2_, Al_2_O_3_ and TiO_2_ (Figure 1C). Note that Figure 1B shows a very small amount of dye self‐decolorization when the dye is illuminated in the absence of a catalyst, while Figure 1C shows no decolorization for several composite materials. We hypothesize that these Co_3_O_4_‐containing materials absorb most of the light without initiating a decolorization reaction, thereby preventing light absorption by the dye and therefore dye self‐decolorization.

The diffuse reflectance of the Co_3_O_4_‐containing composite materials was measured with UV‐Vis‐NIR diffuse reflectance spectroscopy (DRS). The obtained spectra were converted to F(R_∞_) through the Schuster‐Kubelka‐Munk (S‐K‐M) equation, giving a property proportional to the absorbance of our materials. DRS shows that these materials resulted in similarly shaped diffuse reflectance spectra, with the exception of the material containing zeolite ZSM‐5 (Figure S3). Therefore, cobalt oxide is referred to as Co_x_O_y_ in this ZSM‐5‐composite material. Significant dye decolorization was observed only when Co_3_O_4_ was combined with TiO_2_ or Al_2_O_3_, while no activity was observed for the other combinations. However, Co_3_O_4_/Al_2_O_3_ showed similar activity in light and dark conditions (Figure S4). Therefore, Co_3_O_4_/TiO_2_ was deemed the only material showing photocatalytic activity, and this material was chosen for further in‐depth exploration.

**Figure 2:**
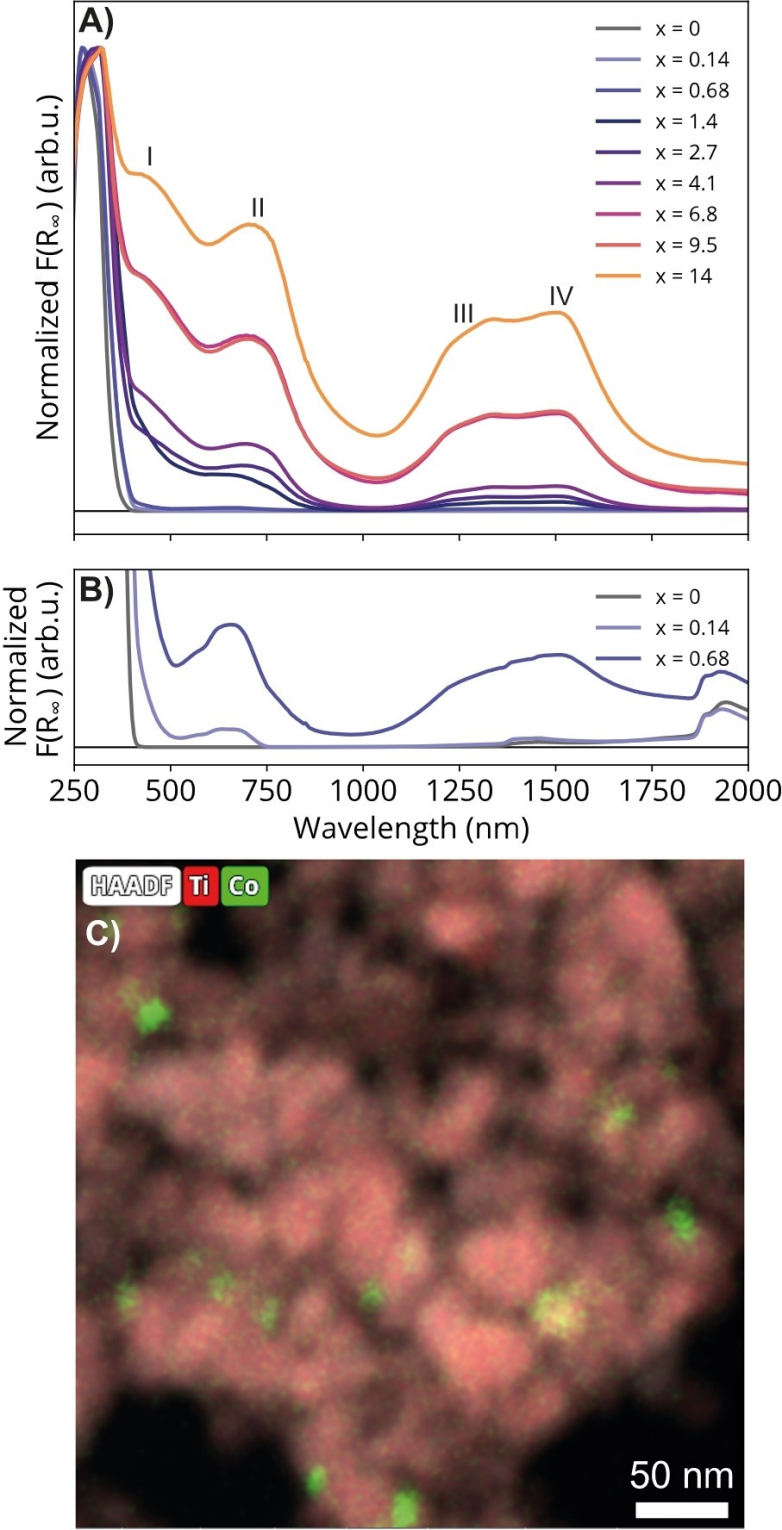
A) Diffuse reflectance spectra of *x* wt % Co_3_O_4_/TiO_2_ catalyst materials, whereby the intensity is converted via the Schuster‐Kubelka‐Munk (S−K‐M) equation and normalized to the TiO_2_ absorption band at 300 nm. Bands I–IV are characteristic of Co_3_O_4_ in the spinel structure. *x*=0 refers to bare TiO_2_. B) Zooming in on the intensity axis. C) Transmission electron microscopy ‐ energy dispersive X‐ray spectroscopy of the 1.4 wt% Co_3_O_4_/TiO_2_ material showed that Co_3_O_4_ particles (green spots) are distributed over the TiO_2_ surface (red). The Co_3_O_4_ particles are approximately 10–30 nm in size as manually determined using the software ImageJ.

Co_3_O_4_/TiO_2_ composite materials were synthesized in different weight loadings, referred to as *x* wt % Co_3_O_4_/TiO_2_, where *x* is the weight percentage of Co_3_O_4_ in the material (0.14 < *x* <14). DRS (Figure 2A) shows that these photocatalyst materials have an absorption band at approximately 300 nm, originating from TiO_2_. F(R_∞_) was normalized to this TiO_2_ absorption band, since we diluted strongly absorbing powders with polytetrafluoroethylene (PTFE) before measuring. All spectra have similar shapes, including the lowest weight loadings (Figure 2B), indicating similar chemical compositions. The absorption bands in the visible (about 400–700 nm) and near‐infrared (1000‐1700 nm) region belong to the mixed‐phase cobalt(II,III) oxide, Co_3_O_4_, in the spinel structure. The broad feature at position I occurs due to *p‐d* charge‐transfer transitions from *p*(O^2−^)→*t*_2_(Co^2+^) and *p*(O^2−^)→*e_g_* (Co^3+^). Band II arises from internal *d‐d* transitions from *t_2g_* (Co^3+^) → *t_2_* (Co^2+^).^[53]^ Band III was assigned to a charge‐transfer between Co^2+^(Π^2^ *t_2_*)→Co^3+^(σ*e). Band IV was assigned to the ^4^A_2_(F)→^4^T_1_(F) crystal field transition.[^54^, ^55^] X‐ray diffraction (XRD) further corroborated the presence of Co_3_O_4_ (Figure S9). Transmission electron microscopy ‐ energy dispersive x‐ray (TEM‐EDX) spectroscopy of the 1.4 wt% Co_3_O_4_/TiO_2_ material showed that Co_3_O_4_ particles were distributed over the TiO_2_ surface (Figure 2C). Using the open‐source software ImageJ, the particles shown in Figure 2C were manually measured to be between 10 and 30 nm in size, which is comparable to the size of the TiO_2_ particles. It should be noted that TEM does not provide information on the electronic (band) structure of the material, *i. e*., the formation of a heterojunction cannot be proven. Techniques such as transient absorption spectroscopy could be employed to gain a better understanding of charge carrier (electrons and holes) dynamics within the composite material. Moreover, ultraviolet photoelectron spectroscopy may elucidate the material's band structure.

**Figure 3:**
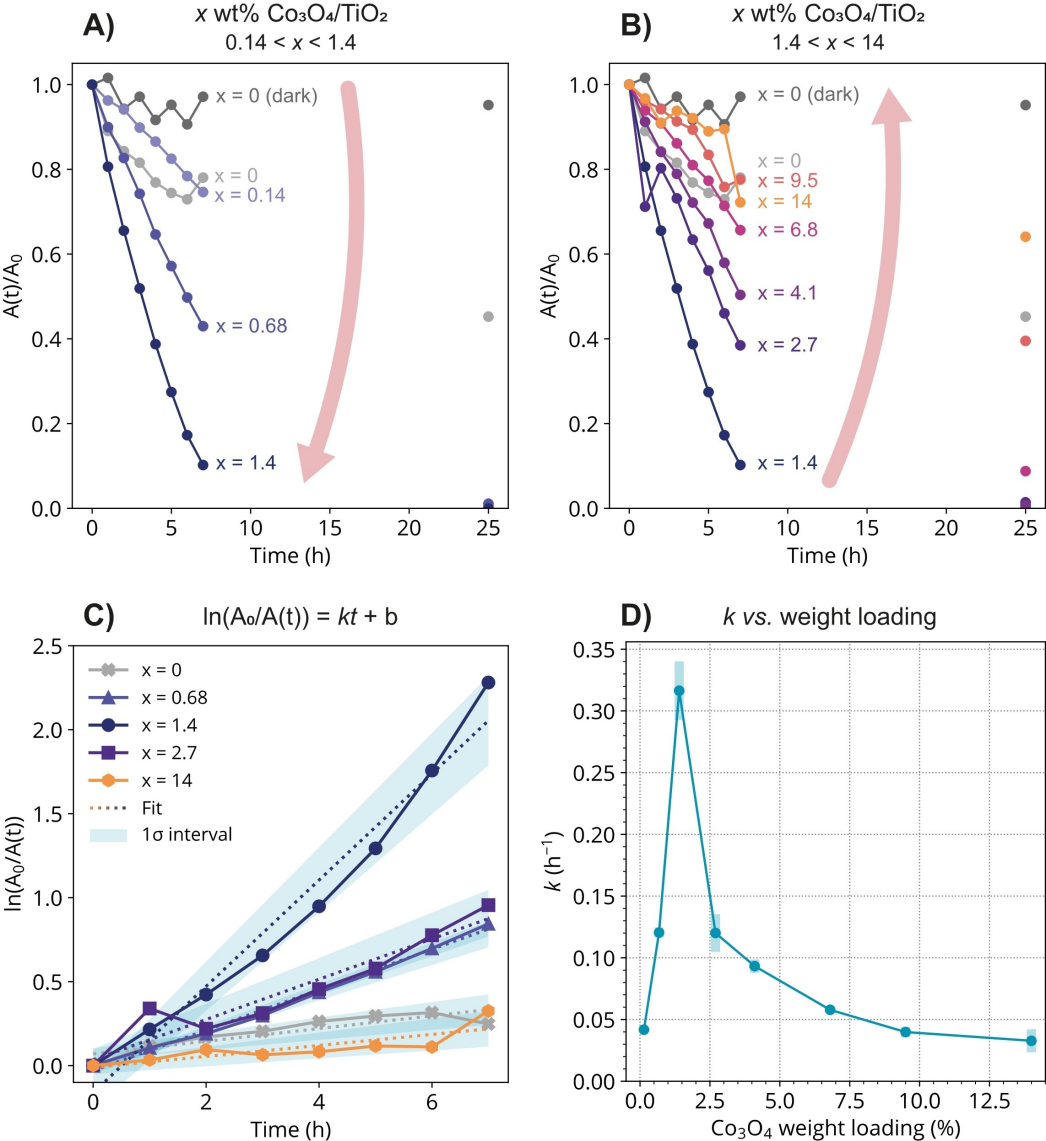
A−B) Indigo carmine dye was decolorized under green light illumination (λ_max_=520 nm) using *x* wt % Co_3_O_4_/TiO_2_ photocatalyst materials, where 0.14 < *x* <1.4 (A) or 1.4 < *x* <14 (B). Bare TiO_2_ (*x*=0) is also shown under light and dark conditions. The normalized absorbance of colored dye molecules is expressed as A(t)/A_0_. The dye absorbance was not measured between 7 h and 25 h. C) Linear fit of ln(A_0_/A(t)) in the form of *kt* + b, where ln(A_0_/A(t)) is the natural logarithm of the normalized dye absorbance after decolorization experiments performed on *x* wt % Co_3_O_4_/TiO_2_. The red dashed line indicates the fit and the shaded areas indicate one standard deviation (σ) around the fit values, as determined from the covariance matrix by taking the square root of the diagonal values. For clarity, some materials were left out to highlight the best‐performing 1.4 wt% Co_3_O_4_/TiO_2_ material. D) Apparent rate constant *k* as a function of Co_3_O_4_ weight loading. The error bars indicate one standard deviation.

We evaluated the photocatalytic activity of Co_3_O_4_/TiO_2_ composite materials for the decolorization of indigo carmine under green LED light illumination (λ_max_=520 nm, 2.4 eV), thereby exploring the optimal Co_3_O_4_ loading as well as the reaction time. Note that the absorption spectrum of TiO_2_ and the emission spectrum of this green lamp do not overlap – the green light energy is insufficient to create electron‐hole pairs in TiO_2_, but selectively excites the Co_3_O_4_. The green LED light also has a minor overlap with the absorption of the dye (Figure S11). Figure 3A shows that the amount of decolorized dye increased when increasing the loading of Co_3_O_4_ on TiO_2_ from 0.14 to 1.4 wt%. When the Co_3_O_4_ loading was increased beyond 1.4 wt%, the amount of decolorized dye decreased again (Figure 3B). These figures show that the 1.4 wt% Co_3_O_4_/TiO_2_ material resulted in the highest degree of dye decolorization rate, with 90 % of the dye being decolorized after 7 h compared to only about 50 % for TiO_2_ only (x=0). The fact that some dye decolorization was observed when using a TiO_2_ photocatalyst under green light, which does not have enough energy to excite TiO_2_, suggests that light absorption by the dye itself plays a role in its decolorization, along with effects of light absorption by Co_3_O_4_ in the Co_3_O_4_/TiO_2_ composite materials.

To gain further insight in the decolorization reaction kinetics, ln(A_0_/A(t)) was plotted as a function of time (Figure 3C). For clarity, we included only the 1.4 wt% Co_3_O_4_/TiO_2_ material due to its excellent performance as determined from Figures 3A−B, two materials with a higher and a lower Co_3_O_4_ weight loading than this 1.4 wt%, the highest Co_3_O_4_ weight loading, and the bare TiO_2_. For first‐order reaction kinetics, ln(A_0_/A(t)) is linearly related to the illumination time *t* through the apparent rate constant *k*: ln(A_0_/A(t))=*kt* + b (see SI section 9).[^56^, ^57^] We fit the ln(A_0_/A(t)) data to a linear function of the form *kt* + b. Figure 3C shows that the highest decolorization rate constant, *i. e*., the largest slope, was found for 1.4 wt% Co_3_O_4_/TiO_2_. ln(A_0_/A(t)) seems to be not completely linear for 1.4 wt% Co_3_O_4_/TiO_2_, indicating that some second order kinetics influence the rate of dye decolorization. ln(A_0_/A(t)) is, however, linear for all other materials tested (Figure S7).

The apparent rate constant *k* was plotted as a function of Co_3_O_4_ weight loading in Figure 3D, which shows that the largest rate constant (*k*=0.32 h^−1^; highest decolorization rate) is observed for 1.4 wt% Co_3_O_4_/TiO_2_, while all other materials showed smaller rate constants.

It should be noted that the initial dye concentration can affect the rate of decolorization. For the range of dye concentrations used in this study (fresh dye concentration of 100 ppm), a higher dye concentration typically leads to an increased decolorization rate due to an increased probability of a dye molecule being in close proximity to reactive oxygen species.[^50^, ^58^] The initial dye concentration differed between reactions performed with different catalysts, because a different amount of dye molecules was adsorbed to the different catalyst surfaces (Figure S5). However, Figure S5 shows that the initial dye concentration is lowest for the decolorization reaction performed with 1.4 wt% Co_3_O_4_/TiO_2_, the material with the highest decolorization rate. Therefore, the 1.4 wt% Co_3_O_4_/TiO_2_ material is still considered the best performing material.

### Detection of Reaction Products by Nuclear Magnetic Resonance

**Figure 4:**
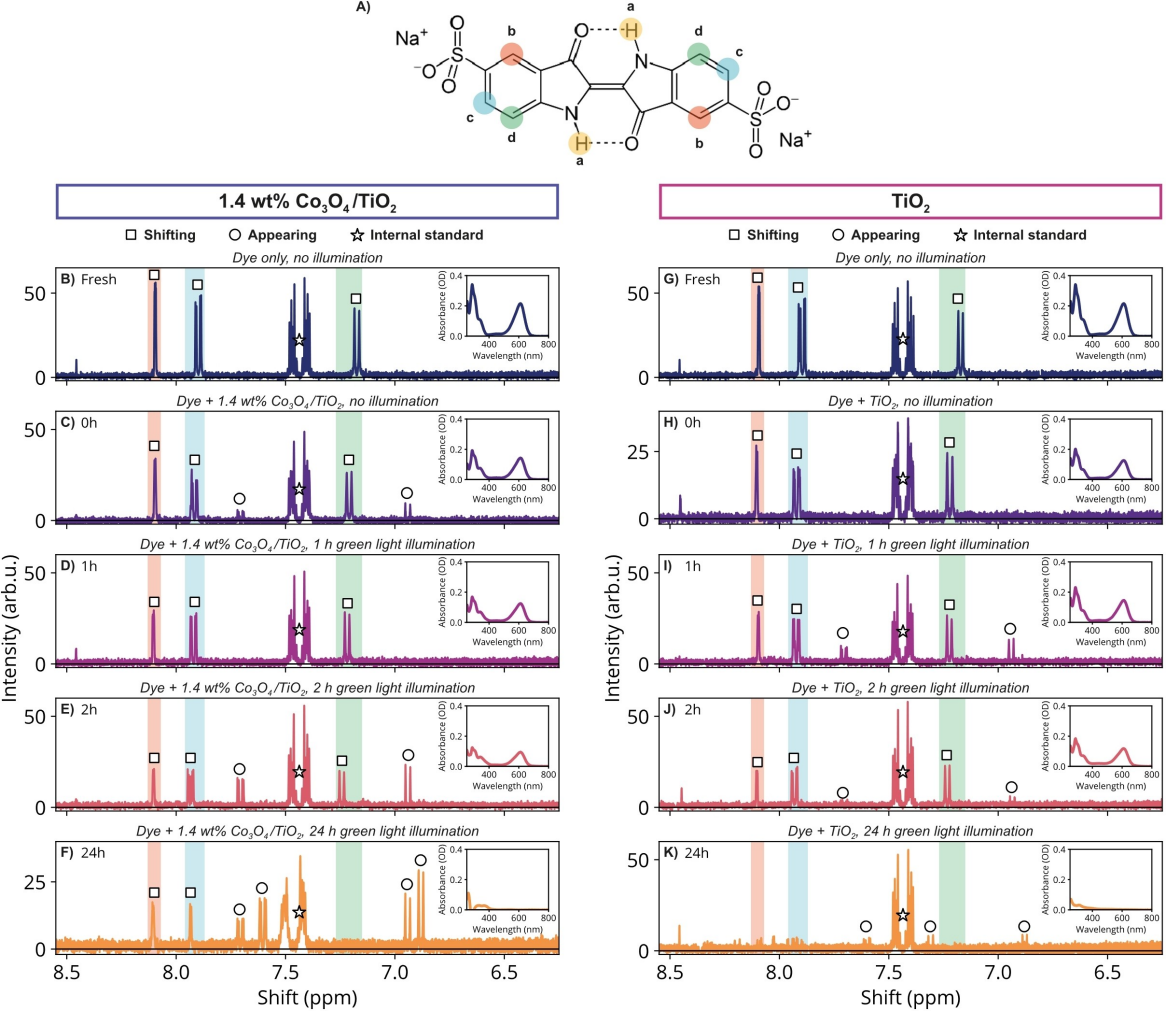
A) Indigo carmine dye molecule. The colored circles indicate which protons atoms result in unique ^1^H‐NMR signals. Since the molecule is symmetrical, only four chemically distinct protons are present. The remaining panels B−K show NMR spectra of the following mixtures: fresh indigo carmine dye (B and G; 100 ppm in D_2_O); indigo carmine dye mixed with 1.4 wt% Co_3_O_4_/TiO_2_ (C−F) or TiO_2_ (H−K), after equilibrating overnight and at 0 h (directly before illumination), 1 h, 2 h and 24 h of green LED light illumination (λ_max_=520 nm), respectively. Several signals are characterized as signals that are either shifting in position on the x‐axis (□) or as appearing over time (○). The signal indicated with a star originates from the internal standard potassium phthalate monobasic. The insets in each panel show the UV‐Vis spectrum correlating to that NMR spectrum. All solutions were diluted 20 times before measuring the UV‐Vis absorbance.

^1^H‐NMR was used to obtain more insights in the decolorization reaction mechanism. To this end, indigo carmine dye was decolorized under green light illumination (λ_max_ = 520 nm) using the 1.4 wt% Co_3_O_4_/TiO_2_ catalyst and bare TiO_2_ catalyst, using D_2_O as the solvent instead of H_2_O to suppress the water signal. Aliquots of the reaction mixture were taken directly before starting illumination, and at 1, 2 and 24 h after starting illumination. NMR spectra were recorded of fresh indigo carmine dye and of each taken aliquot (Figure 4). Before measuring the NMR spectra, a solution of potassium phthalate monobasic in D_2_O was analytically added to each sample as an internal standard, so that the final concentration of the internal standard was 100 ppm. It should be noted that the reaction was performed using the same dye concentration as used for the reactions in Figure 3 (100 ppm). This results in a low‐intensity NMR spectrum with a low signal‐to‐noise ratio. However, we chose to work with these concentrations for NMR measurements to ensure comparability between experiments, since the initial concentration for example can influence the reaction rate.[^50^, ^58^]

Figure 4A shows the indigo carmine dye molecule. Since the molecule is symmetrical, there are four protons (**a**‐**d**, shaded in yellow, red, blue and green, respectively) that give rise to a unique NMR signal. It is expected that proton **a** quickly exchanges with the solvent, so that it is not observed in the NMR spectra. In the remaining panels, the signals shaded in red, blue and green correspond to protons **b**, **c** and **d**, respectively.^[59]^ Figure 4B and G show the same NMR spectrum of the fresh indigo carmine dye as a 100 ppm solution in D_2_O. Indeed, three unique signals are observed corresponding to protons **b**‐**d**. On the left‐hand side of Figure 4 (panel B−F), NMR spectra are shown for the experiment using the 1.4 wt% Co_3_O_4_/TiO_2_ catalyst, while the right‐hand side (panel G−K) shows NMR spectra using the bare TiO_2_ catalyst. The supporting information contains an in‐depth analysis of the NMR data (SI section 14), as well as the relative absorbance of the reaction mixtures over time (Figure S12) and ^1^H‐NMR multiplet assignment (Figures S13 and S14). Signals in Figure 4 indicated with are related to the intact indigo carmine molecule. These signals shift in their position on the x‐axis (chemical shift) over time. Such a shift can have several causes. First, indigo carmine can exist in a cis‐ and a trans‐form.^[60]^ If the central double C=C bond is converted to a single C−C bond or weakened by donation of electrons into the antibonding orbital for example, some trans‐indigo carmine might be converted to cis‐indigo carmine (or vice versa), causing a shift in NMR signals. Another explanation might be that the nitrogen atom is deprotonated, leaving a (partial) negative charge on the nitrogen, which influences the shielding of nearby protons (**b**‐**d**) attached to the benzene ring. If a quick exchange exists between two states of the molecule (*e. g*., cis‐/trans‐form, protonated/deprotonated form), this can be expressed in the NMR spectrum as a shifting signal that is a (weighted) average of two separate signals that are close together. It should be noted that the signals, most notably those of protons **c** and **d**, have already shifted after the dye has been stirred in the dark in the presence of the catalysts, as seen when comparing the spectra of the fresh dye to the spectra at t=0 h.

The signal area normalized to the internal standard can provide information about the chemical decomposition of the dye molecule, since these signals originate from the intact dye molecule. The normalized area of protons **b–d** all decrease following the same trend (Figure S13F and Figure S14F). Additionally, the decreases in NMR signal area and those in UV‐Vis absorbance match, both indicating a decrease of ~35 % for 1.4 wt% Co_3_O_4_/TiO_2_ and of ~10‐20 % for TiO_2_ after 2 h. In both cases, the shape of the UV‐Vis spectra changes similarly over time, and the dye was fully decolorized after 24 h, as indicated by the UV‐Vis absorption spectra (Figure S12 and insets in Figure 4). However, the NMR data shows that the reaction mixture has a different composition after 24 h when using the two different catalysts to decolorize indigo carmine.

Signals in Figure 4 indicated by ○ are signals that appear as the decolorization reaction progresses. The multiplets are assigned in Figures S13 and S14. These appearing signals are related to degradation products of the indigo carmine molecule. For 1.4 wt% Co_3_O_4_/TiO_2_, some products already seem to be present after equilibration in the dark (Figure 4C), disappear again at t=1 h (Figure 4D), and return and remain until t=24 h (Figure 4E−F), along with additional signals appearing at t = 24 h. Moreover, the signals at t=2 h and 24 h in the red and blue shaded areas (proton **b** and **c**) could originate from reaction products, since the signal of proton **d** has disappeared after 24 h. This suggests that the indigo dye molecule is not fully chemically degraded after 24 h of reaction time, while it is fully decolorized as indicated by UV‐Vis. For TiO_2_, newly appearing signals are first observed at t=1 h. These signals appear at the same positions as those appearing for the 1.4 wt% Co_3_O_4_/TiO_2_ catalyst and seem to significantly reduce after t=1 h. Additionally, the originally present signals shaded in red, blue, and green have completely disappeared after 24 h, while some reaction products seem to remain.

The interpretation of the remaining, decreasing and appearing NMR signals for the decolorization reaction performed using a 1.4 wt % Co_3_O_4_/TiO_2_ catalyst indicates that the indigo carmine molecule is not degraded completely. After 24 h, some newly appearing signals were observed compared to the fresh dye, indicating that some reaction products remain in solution. Some signals originating from the intact indigo carmine molecule remain visible after 24 h, indicating that at least part of the molecule stays intact. Since the coupling constant of *J*=8.6 Hz was also observed for newly appearing signals, it is expected that the reaction products are structurally similar to the intact dye molecule, *e. g*., protons attached to an aromatic ring, while having a different chemical environment as indicated by their differing chemical shift (position on x‐axis), *e. g*., different substituents on that aromatic ring. However, it remains difficult to determine specific reaction products based on these data alone.

When a TiO_2_ catalyst was used, some of the same reaction products were observed as for the 1.4 wt% Co_3_O_4_/TiO_2_ catalyst between 0 and 2 h of reaction time. Again, determining which reaction products are formed remains difficult. However, the NMR spectra showed no reactants or products were left in the reaction mixture, indicating that the indigo carmine molecule was completely degraded into volatile products or into products invisible in ^1^H‐NMR (*i. e*., products containing no H‐atoms).

The interpretation of both sets of UV‐Vis and NMR data indicates that observations from NMR give complementary insights to the UV‐Vis data. While both catalysts resulted in the disappearance of (nearly) all absorption bands, they resulted in significantly different NMR spectra after 24 h. The UV‐Vis absorption band at 611 nm originates from the chromophore group, *i. e*., the central C=C bond surrounded by two ‐NH donor groups and two C=O acceptor groups.[^23^, ^61^] This band decreases in intensity at a different rate than the absorption bands below 400 nm, which mainly belong to the (substituted) benzene, C=O, and ‐NH groups present in indigo carmine.[^62^, ^63^] This indicates that the chromophore group decolorizes (*i. e*., decreases in absorbance) before the other mentioned groups are affected. This suggests that the molecule photodegrades into the smaller building blocks, which still contain for example some (substituted) benzene, C=O, and/or ‐NH groups. Moreover, it is known that so‐called auxochrome groups, which are substituents to the chromophore group, can enhance the color of the chromophore group.[^64^, ^65^, ^66^] However, these auxochrome groups are not necessarily visible in NMR. If these auxochrome groups are detached, the absorption spectrum of the analyte can change differently than the NMR spectrum, for example resulting in a shift in signals or in new signals appearing. Decomposition of the chromophore group itself, *e. g*., breakage of the central C=C bond, can result in changes of both the UV‐Vis and NMR spectrum. However, we cannot exclude that the dye could also decolorize due to an electron residing on the dye molecule after it is excited by the used light source. We hypothesize that the auxochrome groups are detached from the dye molecule during the photocatalytic reaction first, resulting in an absorbance change, and that any reaction products can then degrade further, resulting in a changing NMR spectrum. The NMR data suggests that 1.4 wt% Co_3_O_4_/TiO_2_ catalyst does not fully chemically decompose the dye molecule, while the molecule does seem to be almost completely degraded when TiO_2_ was used, even though the UV‐Vis spectra (indicating decolorization) look very similar for both catalysts.

### Proposed Catalyst Band Structure

The decolorization of indigo carmine was determined with UV‐Vis spectroscopy and compared to the NMR data to gain more insight in the decolorization and/or degradation mechanism. For the two catalyst materials on which the NMR experiments were performed under green light illumination (*i. e*., 1.4 wt% Co_3_O_4_/TiO_2_ and TiO_2_), the NMR spectra looked significantly different. TiO_2_ showed a high degree of decomposition of the dye molecule, showing almost no reactant dye molecules or reaction products in the NMR data, while 1.4 wt% Co_3_O_4_/TiO_2_ resulted in some degradation products that were still present in the reaction mixture after 24 h reaction time. While the NMR spectra showed differences, the UV‐Vis spectra looked very similar, *i. e*., both dyes were completely decolorized after 24 h. Therefore, we hypothesize that the decolorization/degradation mechanism is different on these two catalysts.

**Figure 5:**
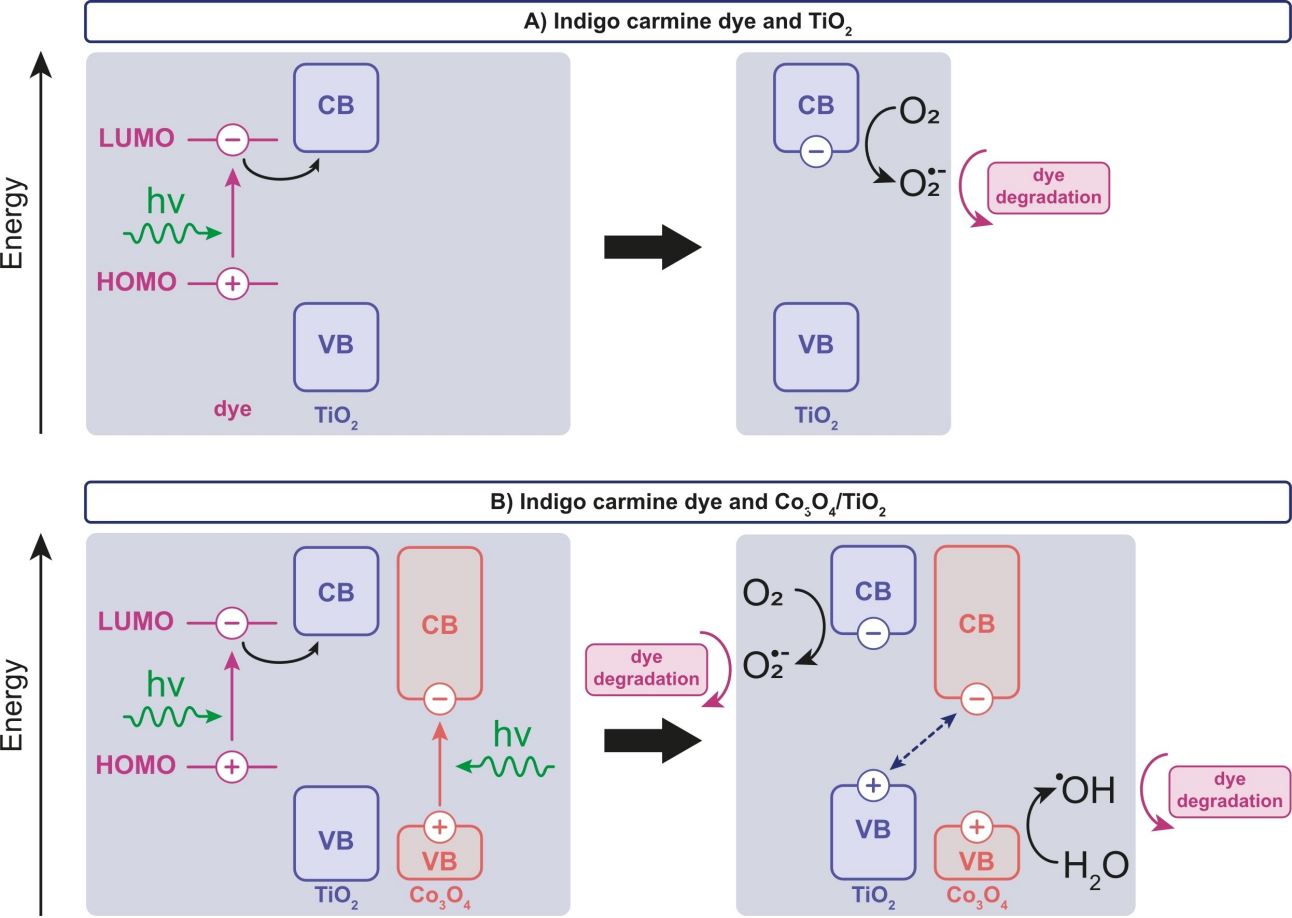
Suggested dye decolorization mechanisms for indigo carmine dye in the presence of A) a TiO_2_ catalyst, and B) a Co_3_O_4_/TiO_2_ catalyst.

Based on the dye decolorization trends shown in Figure 3, combined with the NMR and UV‐Vis spectra of Figure 4, we propose a mechanism, as outlined in Figure 5. Since Figure 3 shows that TiO_2_, a material that only absorbs UV light, can also decolorize the dye molecule under green light illumination, we hypothesize that the dye aids in its own decolorization. Based on the band edge positions of TiO_2_ and HOMO/LUMO positions of indigo carmine,[^27^, ^60^, ^67^] we propose that an electron is excited from the dye's highest occupied molecular orbital (HOMO) to the lowest unoccupied molecular orbital (LUMO), after which hole transfer takes place to the TiO_2_ catalyst (Figure 5A). A radical oxygen species O_2_^•–^ is then formed, which can subsequently react with the dye molecule, causing its degradation. In the case where a Co_3_O_4_/TiO_2_ catalyst is used (Figure 5B), we hypothesize that the process shown in Figure 5A may be combined with the creation of a heterojunction between TiO_2_ (n‐type) and Co_3_O_4_ (p‐type).[^24^, ^68^, ^69^] Based on the available literature, the conduction band (CB) and valence band (VB) edges of TiO_2_ were estimated to be about −0.4 and +2.8 V vs. NHE, respectively, while the CB and VB edges of Co_3_O_4_ were estimated at about +0.5 and +2.4 V vs. NHE.[^27^, ^68^, ^69^, ^70^, ^71^, ^72^, ^73^, ^74^, ^75^] This band alignment would mean that no transfer of charge carriers can take place from Co_3_O_4_ to TiO_2_. However, in this work we observed that adding a small amount of Co_3_O_4_ to TiO_2_ has a significantly positive effect on the dye decolorization rate. Therefore, we propose the band alignment shown in Figure 5B, which might be a result of, *e. g*., band bending/shifting after heterojunction formation.^[76]^ We propose that an electron is excited in Co_3_O_4_, after which the created hole results in the formation of ^•^OH radicals, resulting in the degradation of the dye molecule. Excited CB electrons in Co_3_O_4_ and VB holes in TiO_2_ can recombine. The combination of the two processes in the Co_3_O_4_/TiO_2_ catalyst material possibly enhances the decolorization rate of indigo carmine compared to the bare TiO_2_ catalyst material. Since an optimal weight loading of 1.4 wt% Co_3_O_4_ on TiO_2_ was observed, we hypothesize that only a small amount of Co_3_O_4_ is needed for light absorption, as shown in Figure 5B, while an excess amount of Co_3_O_4_ slows down the decolorization process due to less TiO_2_ surface availability for the creation of reactive oxygen species. Another option is that the degradation process is slower on Co_3_O_4_/TiO_2_ than on bare TiO_2_, for example due to a decreased charge mobility in Co_3_O_4_.

## Conclusions

Co_3_O_4_ composite materials with six different zeolite and oxide materials were synthesized to determine their ability to decolorize indigo carmine dye under solar light illumination. Co_3_O_4_/TiO_2_ was the overall best performing catalyst for this reaction among the different materials tested. XRD and DRS showed that cobalt oxide was present as mixed‐phase cobalt(II,III) oxide (Co_3_O_4_). Co_3_O_4_ was combined with TiO_2_ in weight loadings ranging from 0.14 to 14 wt% by means of a wet impregnation method. Indigo carmine dye was decolorized on all Co_3_O_4_/TiO_2_ materials, where weight loadings below 6.8 wt% showed a higher decolorization rate constant than bare TiO_2_ under green light illumination, indicating a positive effect of Co_3_O_4_ on the photocatalytic activity of TiO_2_. By taking the natural logarithm of the normalized dye absorbance (A(t)/A_0_) and fitting the data to a linear formula, the apparent rate constant *k* was determined to be the largest for 1.4 wt% Co_3_O_4_/TiO_2_, giving it the highest rate of dye decolorization. UV‐Vis spectroscopy combined with NMR suggests that the auxochromic substituent groups of the indigo carmine molecule are altered or detached first, possibly combined with the decomposition of the chromophore group itself, after which further degradation can take place. NMR showed that degradation products can be formed during the decolorization reaction, indicating that chemical decomposition is at least partially the cause for the observed dye decolorization. Importantly, UV‐Vis spectroscopy and NMR are complementary techniques for the analysis of the dye decolorization reaction. They showed that decolorization of indigo carmine dye is not necessarily due to chemical decomposition alone, as UV‐Vis spectroscopy showed that the dye was completely decolorized for 1.4 wt% Co_3_O_4_/TiO_2_, while NMR showed signals belonging to some (degradation) products as well as to the intact dye molecule. Therefore, we suggest that one should be cautious in drawing conclusions with respect to dye decolorization *vs*. dye degradation.

Both under green light and simulated solar light, 1.4 wt% Co_3_O_4_/TiO_2_ shows a higher dye decolorization rate than bare TiO_2_, indicating that Co_3_O_4_ has a positive effect on the decolorization rate at this weight loading. However, since the rate seems to be optimal at 1.4 wt%, it is not expected that Co_3_O_4_ itself is photo‐catalyzing the reaction, but rather that Co_3_O_4_ acts as a co‐catalyst, giving TiO_2_ the ability to utilize visible light for photocatalytic decolorization reactions. A band structure is proposed where the dye molecule absorbs the light and thus aids in its own degradation when TiO_2_ is used as the catalyst. When a Co_3_O_4_/TiO_2_ catalyst is used, Co_3_O_4_ absorbs the light and acts as the co‐catalyst to TiO_2_ for degradation of the dye molecule, in addition to the dye self‐degradation process taking place.

Future experiments will be directed towards using TiO_2_‐supported Co_3_O_4_ for photocatalytic oxidation reactions of gaseous VOCs under visible LED light illumination. The analysis of the NMR data, complemented by the UV‐Vis data, could be improved by performing 2D‐NMR experiments (*e. g*., H−H coupling or H−C coupling experiments) and/or combining NMR with liquid chromatography‐mass spectrometry (LC–MS) for determining reaction products in more detail. Additionally, density functional theory studies and transient absorption spectroscopy could provide valuable insights into the photocatalytic dye decolorization mechanism over our Co_3_O_4_/TiO_2_ photocatalyst. By using DFT to accurately calculate the conduction‐ and valence band edges of Co_3_O_4_ and TiO_2_, both in their isolated and combined form, as well as the HOMO and LUMO levels of indigo carmine in relation to the (composite) photocatalyst, the band alignment could be elucidated. Transient absorption spectroscopy could aid in determining how the charge carriers participate in the redox reactions required for ROS creation, and thus how Co_3_O_4_ and TiO_2_ contribute to the dye decolorization and/or degradation. In addition, ultraviolet spectroscopy could elucidate the band structure of the composite Co_3_O_4_/TiO_2_ material.

## Supporting Information

The authors have cited additional references within the Supporting Information.[^77^, ^78^, ^79^, ^80^]

## Author Contributions

M.E. de G. conducted and analyzed the experiments and wrote the paper with input from E.M.H. and B.M.W. N.G. synthesized Co_3_O_4_/zeolite and Co_3_O_4_/oxide composite materials and performed dye decolorization experiments with these materials. B.K. assisted in performing NMR experiments and NMR data interpretation. R.L.G. added valuable insights to the band structure and alignment of Co_3_O_4_, TiO_2_ and composites thereof through a literature study, and performed TEM‐EDX measurements. J.F., E.M.H. and B.M.W. supervised the project.

## Conflict of Interests

The authors declare no conflict of interest.

1

## Supporting information

As a service to our authors and readers, this journal provides supporting information supplied by the authors. Such materials are peer reviewed and may be re‐organized for online delivery, but are not copy‐edited or typeset. Technical support issues arising from supporting information (other than missing files) should be addressed to the authors.

Supporting Information

## Data Availability

The data that support the findings of this study are available from the corresponding author upon reasonable request.
