# Supplementary material for: Mechanistic Insights into the Photocatalytic Indigo Carmine Dye Decolorization by Co3O4/TiO2
Source: Chemphyschem. 2025 Mar 3;26(8):e202400688. doi: 10.1002/cphc.202400688 (PMC12005126; doi:10.1002/cphc.202400688)
Supplement: Supplementary file 1 — Supporting Information [file CPHC-26-e202400688-s001.pdf]

ChemPhysChem

Supporting Information

Mechanistic Insights into the Photocatalytic Indigo Carmine Dye Decolorization by Co_3O_4/TiO_2

Mirjam E. de Graaf, Nejc Godec, Bram T. Kappé, Roos L. Grote, Jitte Flapper, Eline M. Hutter, and Bert M. Weckhuysen*

Supporting Information

Contents

1. TiO_2-based materials for photocatalytic pollutant removal reactions: review and comparison....... 2
2. Experimental methods 3
3. Wt% Co to wt% Co_3O_4 conversion 4
4. Dye decolorization: data processing 5
5. Composite materials of M_xO_y (M = Fe, Co, Mn) and TiO_2 5
6. Co_xO_y composite materials with different zeolites and oxides 6
   UV-Vis-NIR diffuse reflectance spectroscopy measurements 6
   Dye decolorization: selecting the best performing material 7
7. Dye decolorization: absolute absorption values 9
8. Effect of light on dye when there is no catalyst present 9
9. Dye decolorization: fitting data 10
10. Dye decolorization: solar versus green light 10
11. X-ray diffraction measurements 11
12. Transmission electron microscopy measurements 12
13. Lamp emission spectra versus dye absorption spectrum 12
14. Dye degradation products detected by nuclear magnetic resonance 13
   General observations on NMR data 14
   1.4 wt% Co_3O_4/TiO_2 14
   TiO_2 15

1. TiO_2-based materials for photocatalytic pollutant removal reactions: review and comparison

Table S1. Overview of some composite photocatalyst materials used for indoor pollutant removal referenced in this work. The Co_3O_4/TiO_2 materials investigated in this work are also included in this table on the bottom row.

**Catalyst composition**	**Weight loading**	**Type of reaction**	**Illumination conditions**	**Reaction rate constant (k)**	**Detection method(s)**	**Reference**
g-C_3N_4/TiO_2	4.1 - 7.0 wt% g-C_3N_4	Methylene blue decolorization	365 nm, 44 W	$0.7 < k_{app} < 1.0$ h^{-1}	UV-vis absorption	3
"	"	"	420 nm, 44 W	$2.1 < k_{app} < 2.7$ h^{-1}	"	"
"	"	"	AM1.5G (sol. sim.), 300 W Xe lamp	$1.5 < k_{app} < 2.3$ h^{-1}	"	"
Fe_2O_3/TiO_2	0.2 - 20 wt% Fe_2O_3	Rhodamine B decolorization	Visible light (Philips TL 8W/54−7656 bulb lamps)	Not reported explicitly (only % degradation; $C/C_0 \approx 0.3$ after 180 min for 2 wt% Fe_2O_3)	UV-vis absorption	10
Co_3O_4/TiO_2	55 wt% Co ≈ 75 wt% Co_3O_4	Methyl orange decolorization	Ordinary visible light (no spectral information provided)	$k_1 = 0.0097$ min^{-1} = 0.58 h^{-1}	UV-vis absorption	18
Co_3O_4/TiO_2	85, 90, 95, 98 wt% Co_3O_4	Methyl orange decolorization	- (reaction via peroxymonosulphate)	k = 0.166 min^{-1} = 10.0 h^{-1}	UV-vis absorption	19
Co_3O_4 quantum dots/TiO_2 nanobelt	2, 4, 6 wt% Co_3O_4	Water splitting	Sol. sim. (300 W Xe lamp with AM1.5 filter)	H_2 evolution: 41.8 µmol h^{-1} g^{-1}<br>O_2 evolution: 22.0 µmol h^{-1} g^{-1}	Gas chromato-graphy	20
Co_3O_4 nanodots/ TiO_2 nanosheets	Only reported amount of Co-precursor added (20-60 mg)	Enrofloxacin degradation	Xe lamp, 500 W	$0.75 < k < 1.75$ h^{-1}	High-performance liquid chromato-graphy (HPLC)	24
Co_3O_4/TiO_2 nanotubes	Not reported	Methyl orange decolorization	365 nm, ~15 mW/cm^2	Not reported explicitly (only % degradation; $C/C_0 \approx 0.5$ after 90 min)	UV-vis absorption	73
Co_3O_4/TiO_2	1.4 wt% Co_3O_4	Indigo carmine decolorization	520 nm, 5.2 mW/cm^2	k = 0.32 h^{-1}	NMR & UV-vis absorption	*This work*
"	0.68, 2.7 wt% Co_3O_4	"	"	k_{app} ~ 0.12 h^{-1}	"	"
"	0.14, > 4.1 wt% Co_3O_4	"	"	$k_{app} < 0.1$ h^{-1}	"	"

Table S1 shows that the way in which reaction conditions and results are reported may vary between different research works. For example, the reaction rate constant is not always reported explicitly, and some light source irradiances and weight loadings are not reported completely. Therefore, comparing between different research works is not trivial. In addition, this table shows that the dye decolorization over our Co_3O_4/TiO_2 materials were analyzed by means of two complementary techniques, namely UV-Vis absorption spectroscopy and nuclear magnetic resonance spectroscopy. This unique combination of techniques not only provides insight into the decolorization of the dye, but also on its chemical decomposition, as the two are not necessarily identical.

2. Experimental methods

Synthesis of Co_xO_y composite materials. Composite materials of Co_xO_y and six different zeolite or oxide materials were synthesized using a wet impregnation method. The used materials were zeolite Beta (NH_4-form, Si/Al = 12, Zeolyst CP814E*), zeolite Y (H-form, Si/Al = 2.6, Zeolyst CBV600), zeolite ZSM-5 (NH_4-form, Si/Al = 11.5, Zeolyst CBV2314), Al_2O_3 (*γ*-phase, >99%, Thermo Fisher Scientific), SiO_2 (high-purity, Davisil Grade 12, pore size 22 Å, Sigma-Aldrich), and TiO_2 (Aeroxide P25, Acros Organics).

The intended weight loading was 3 wt% Co for each composite material. Note that a loading of only Co is assumed here, and not a loading of cobalt oxide, since it was not yet known if the same cobalt oxidation state would be formed for each combination of materials (*i.e.*, the same *x* and *y* in Co_xO_y). In general, several mL of an aqueous solution containing 0.153 g $Co(NO_3)_2{\cdot}6H_2O$ (0.526 mmol; 99% purity, Thermo Fisher Scientific) were added to a glass vial containing 1.0 g of the zeolite or oxide. The mixture was heated to 80°C on a heating plate with an aluminum heating block while stirring. When stirring was no longer possible due to water evaporation, the mixture was further dried overnight in an 80°C static air oven. The resulting powder was ground to a fine powder and dried at 150°C for 90 min followed by calcination at 550°C for 4 h, both using a ramp of 2°C/min.

In addition, photocatalyst powders with different weight loadings of Co_3O_4 on TiO_2 were prepared (*x* wt% Co_3O_4/TiO_2, where the weight loading of Co_3O_4 is $0.14 < x < 14$). Note that a weight loading of Co_3O_4 is used here, since the oxidation state of the cobalt ions was determined through UV-Vis spectroscopy and X-ray diffraction (XRD) (see paragraphs below and SI Section 3). In general, several mL of an aqueous solution containing the appropriate amount of $Co(NO_3)_2{\cdot}6H_2O$ were added to a glass vial containing 1.0 g TiO_2. The same procedure as described above was employed.

UV-Vis-NIR diffuse reflectance spectroscopy (DRS). The absorption spectrum of all synthesized Co_xO_y composite materials was determined by measuring the diffuse reflected light in the UV-Vis-NIR region of the spectrum using a Lambda 950S UV-Vis-NIR spectrophotometer (PerkinElmer) equipped with a deuterium and a halogen light source, a PMT detector (UV-Vis) and InGaAs detector (NIR), and a 150 mm integrating sphere coated with Spectralon®. Polytetrafluoroethylene (PTFE, 35 µm, Sigma-Aldrich) was used as the reference material. Strongly absorbing powders were diluted by mixing them with PTFE and grinding. The diffuse reflectance (R_∞) was converted to a property F(R) that is proportional to the absorbance using the Schuster-Kubelka-Munk (SKM) equation[77]: $F(R) = \frac{(1-R_\infty)^2}{2R_\infty}$.

Transmission electron microscopy - energy dispersive x-ray (TEM-EDX) spectroscopy. Transmission electron microscopy investigations were performed in a Talos F200 microscope operating at 200 kV. The composite material was suspended in ethanol and deposited on a holey carbon/copper 300 mesh grid. Elemental mapping was performed using a high-brightness field emission gun (X-FEG) and a Super-X G2 EDX detector. Particle sizes were manually determined using the software ImageJ.

X-ray diffraction (XRD). Materials were characterized on a Bruker D2 Phaser powder X-ray diffractometer equipped with a Cu K_α X-ray tube (λ = 1.5418 Å). For increased resolution, the 1.4 and 4.1 wt% Co_3O_4/TiO_2 materials were characterized overnight on a Bruker D8 Advance powder X-ray diffractometer equipped with a Cu K_α X-ray tube (λ = 1.5418 Å).

Dye decolorization. 10 mL of a 100 ppm (100 mg/L) indigo carmine (analytical reagent, VWR Chemicals) solution in deionized water was mixed with 100 mg catalyst (resulting in 10 mg catalyst per mL) in a 20 mL glass vial under stirring. The mixture was left to equilibrate overnight in the closed vial under stirring and under dark conditions. For the dye decolorization experiments, the vial was illuminated from the side with a solar simulator (A1-LA125 LightLine Fiberized Solar Simulator (ScienceTech) equipped with an AM1.5G filter, total absolute irradiance of 1 Sun (100 mW/cm^2 at a power setpoint of 83.5%), distance to liquid = 14 cm) or green LED lamp (Eurolite, LED IP FL-30 SMD, 30 W, λ_{max} = 520 nm, distance to liquid = 14 cm, total absolute irradiance = 5.2 mW/cm^2 at 14 cm working distance) while stirring. Aliquots of 300 µL were taken in 60 min intervals and diluted ten times with deionized water. The photocatalyst powder was separated from the liquid by centrifugation at 2000 rpm for 5 min. The supernatant was added to a 1 cm quartz cuvette. The absorption spectrum was

measured between 200-800 nm using a Cary 60 UV-Vis spectrometer (Agilent Technologies) equipped with a Xenon flash lamp.

For the dye decolorization reactions that were analyzed with nuclear magnetic resonance, the reaction was carried out in deuterium oxide (D_2O, 99.90% D, VWR) instead of deionized water. 1.5 mL fresh indigo carmine dye solution (100 ppm in D_2O) was kept for NMR analysis. 8.5 mL of the dye solution was mixed with 85 mg of the 4.1 wt% Co_3O_4/TiO_2 catalyst in a 20 mL glass vial under stirring. The mixture was left to equilibrate overnight in the closed vial under stirring and under dark conditions. 1.5 mL aliquots were collected at t = 0 h (right before illumination), t = 1 h and t = 2 h under simulated solar light illumination. After centrifugation at 2000 rpm for 5 min, the supernatant was isolated. 200 µL of each supernatant was diluted ten times with D_2O and UV-Vis spectra were recorded between 200-800 nm. For NMR analysis, 50 µL of a 0.525 mg/mL solution of potassium phthalate monobasic (standard for quantitative NMR TraceCERT®, Supelco®, VWR Chemicals) in D_2O was added to 700 µL of the undiluted supernatant. The eventual concentration of potassium phthalate monobasic corresponds to a similar molarity as that of the 100 ppm indigo carmine dye.

Nuclear magnetic resonance (NMR). ^1H (400 MHz) NMR spectra were recorded on a Varian AS400 spectrometer at 25 °C using borosilicate glass NMR tubes. Chemical shifts are reported in ppm relative to D_2O. A solution of potassium phthalate monobasic was used as internal standard. The recorded NMR spectra were corrected (phase correction, baseline correction, correction for chemical shift of D_2O) using the software MestReNova 14.2.0.

3. Wt% Co to wt% Co_3O_4 conversion

The mass of the zeolite or oxide (m_{sup}) and weight percentage of Co (x_{Co}; note that this percentage relates to the cobalt ions and not to cobalt oxide) based on the total mass of catalyst (m_{tot}) were first determined. Generally, m_{sup} = 1.0 g and $0.1 < x_{Co} < 10$. The total catalyst mass is made up of the support mass and the Co mass: $m_{tot} = m_{sup} + m_{Co} = m_{sup} + \frac{x}{100} m_{tot} = \frac{m_{sup}}{1 - \frac{x_{Co}}{100}}$. The mass of cobalt needed for a certain x_{Co} can be determined using the total mass: $m_{Co} = \frac{x_{Co}}{100} m_{tot}$. This mass is converted to the mol amount using the molar weight ($M_{w,Co}$ = 58.9 g/mol). Since 1 mol $Co(NO_3)_2{\cdot}6H_2O$ precursor ($M_{w,precursor}$ = 291 g/mol) contains 1 mol Co, this mol amount can be used to calculate the mass of precursor needed. This mass was dissolved in a few mL of deionized water to form the precursor solution.

The weight percentages x_{Co} were converted from wt% Co to wt% Co_3O_4 using the molar mass fraction of Co in Co_3O_4: $\frac{3M_{w,Co}}{M_{w,Co_3O_4}} = \frac{3 \cdot 58.9}{240.8} = 0.734$. Therefore, 1 wt% Co corresponds to $\frac{1}{0.734}$ = 1.36 wt% Co_3O_4. All conversions are shown in Table S2.

Table S2. Conversion of weight percentage Co to weight percentage Co_3O_4.

wt% Co	wt% Co_3O_4	wt% Co_3O_4, rounded
0.1	0.136	0.14
0.5	0.681	0.68
1	1.36	1.4
2	2.73	2.7
3	4.09	4.1
5	6.81	6.8
7	9.54	9.5
10	13.6	14

4. Dye decolorization: data processing

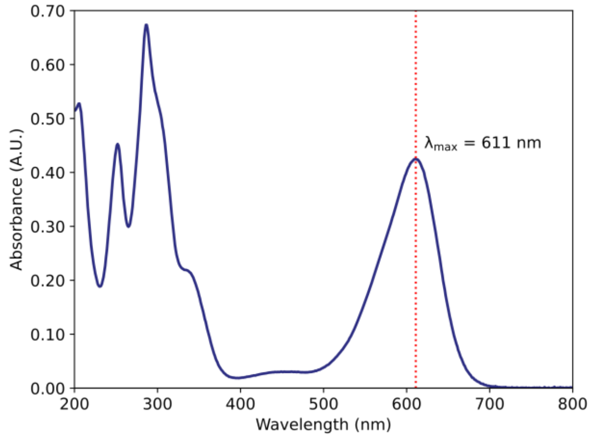

Figure S1. Absorption spectrum of 10 ppm indigo carmine dye.

Figure S1 shows the absorption spectrum of a fresh solution of indigo carmine dye in water. A straight baseline background correction was performed by taking the mean of the absorbance values between 720 and 800 nm and subtracting this value from the whole spectrum, resulting in a straight-baseline correction. Then, the absorption value is determined at a fixed position of 611 nm, as indicated by the red dotted line. The degree of decolorization can be expressed by normalizing the absorbance at a time t > 0 (A(t)) to the absorbance at time t = 0 (A_0). This procedure was applied to all dye decolorization experiments performed in this work to determine A(t)/A_0.

Absorbance relates directly to concentration through the Beer-Lambert law ($A = \varepsilon c l$, where ε is the absorption coefficient, *c* is the concentration and *l* is the path length). However, we deliberately express the degree of decolorization in terms of absorption values, as dye decolorization does not necessarily equate to dye degradation, *i.e.,* chemical decomposition of the molecule. This is further explained in Section 14.

5. Composite materials of M_xO_y (M = Fe, Co, Mn) and TiO_2

A set of screening experiments was performed using composite materials of several metal oxides (M_xO_y) with TiO_2, further denoted as M_xO_y/TiO_2, where M = Mn, Fe or Co. These three metal oxides were chosen, since these metal oxides are among the few that naturally possess a band gap in the visible range, *i.e.*, 1.8 < E_g < 3.1 eV.[27,78] While (nanoparticle) metals and metal oxides have been previously researched extensively in terms of their biological toxicity,[79,80] it should be noted that none of the metals or metal oxides researched in this work are volatile. Nuclear magnetic spectroscopy (NMR) of the dye solution mixed with Co_3O_4/TiO_2 in the paramagnetic range did not show any leaching of cobalt ions (see SI section 14).

The M_xO_y/TiO_2 composite materials were used to decolorize 100 ppm indigo carmine dye in water under solar light illumination, using 10 mg catalyst/mL dye solution. Co_xO_y/TiO_2 was prepared using the wet impregnation method described in Section 1, so that the final catalyst powder contained 3 wt% Co. Mn_xO_y/TiO_2 and Fe_xO_y/TiO_2 were prepared using an ion exchange method. 1.0 g TiO_2 (Aeroxide P25, Acros Organics) was added to 50 mL of 0.1 M $Mn(CH_3COO)_2{\cdot}4H_2O$ (>99%, Sigma-Aldrich) or 0.01 M $Fe(NO_3)_3{\cdot}9H_2O$ (Fisher Scientific) in a 50 mL centrifuge tube. The suspensions were stirred for 3 h at 65°C, after which the suspension was centrifuged once (5 min, 2000 rpm). The supernatant was discarded, 50 mL of fresh 0.1 M $Mn(CH_3COO)_2{\cdot}4H_2O$ or 0.01 M $Fe(NO_3)_3{\cdot}9H_2O$ was added and the suspensions were stirred for another 3 h at 65°C. The powder was washed 3 times with deionized water by centrifugation (5 min, 2000 rpm) and dried overnight in a 80°C static air oven. The resulting powder was ground to a fine powder and dried at 150°C for 90 min followed by calcination at 550°C for 4 h, both using a ramp of 2°C/min.

By analysis with inductively coupled plasma - optical emission spectrometry (ICP-OES), the Fe_xO_y material was found to contain 4.55 wt% Fe. The wt% of the metal can be converted to the wt% of metal oxide following the calculation scheme discussed in SI section 2 for Co/Co_3O_4 wt% conversion. X-ray

diffraction showed that Fe was present in the Fe_2O_3 form. Since $2^*M_{w,Fe}/M_{w,Fe2O3}$ = 0.699, 4.55 wt% Fe corresponds to 6.5 wt% Fe_2O_3. The Mn_xO_y material was found to contain 1.38 wt% Mn by ICP-OES. This weight percentage was too low to be detected by X-ray diffraction, and UV-Vis diffuse reflectance measurements only showed a broad absorption feature over the whole wavelength range (200-2000 nm), providing no information on Mn oxidation state. Therefore, the Mn oxidation state was not determined and no Mn_xO_y weight loading was determined.

Figure S2 shows the absorbance A(t) of indigo carmine dye as a function of illumination time (simulated sunlight, AM1.5G), normalized to the initial absorbance A_0. For easier comparison, the data of main text Figure 1B is also included, *i.e.*, dye only under solar light illumination (blue) and decolorization data for the bare TiO_2 photocatalyst (purple) and for the 4.1 wt% Co_3O_4/TiO_2 photocatalyst (pink). When using Fe_xO_y as the photocatalyst (orange), the dye was decolorized at a lower rate than when using pure TiO_2. This indicates that Fe_xO_y negatively affects the photocatalytic activity of TiO_2 in the calculated weight loading of 6.5 wt%, whereas adding 4.1 wt% Co_3O_4 increased the decolorization rate. We do note that, based on Figure 3A-B, the photocatalytic activity of Fe_xO_y/TiO_2 may be increased if the weight loading is decreased. However, based on the data in **Figure S2**, we chose to continue with the Co_3O_4/TiO_2 material. Mn_xO_y was also screened for its potential dye decolorization capacity. However, after adding Mn_xO_y to the dye solution, and equilibrating in the dark overnight, the dye was already completely decolorized under dark conditions. Therefore, this metal oxide is not included in **Figure S2** and is not considered to be photocatalytically active.

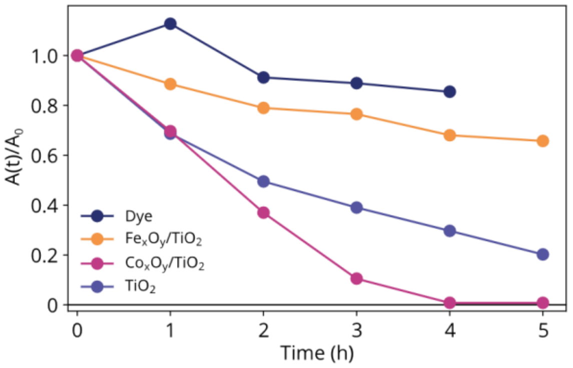

Figure S2. Decolorization of 100 ppm indigo carmine dye in water under simulated solar light illumination, performed with no catalyst present (using only dye and light), composite materials of Fe_xO_y or Co_xO_y and TiO_2, and bare TiO_2.

6. Co_xO_y composite materials with different zeolites and oxides

UV-Vis-NIR diffuse reflectance spectroscopy measurements

Figure S3 shows absorbance spectra of composite materials of Co_xO_y and one of the following: oxides Al_2O_3 or SiO_2, or zeolites Beta, Y or H-ZSM-5, as well as the bare oxides or zeolites. All spectra were normalized to the most intense band in that spectrum. For all Co_xO_y composite materials shown in **Figure S3**, broad absorption bands appear in the visible range (400-700 nm) after addition of Co_xO_y compared to the structure without Co_xO_y. The composite materials of Co_xO_y with Al_2O_3, zeolite Beta, SiO_2, TiO_2 and zeolite Y (**Figure S3A-E**) have similar shapes, with intense bands appearing between 300-700 nm and between 1100-1600 nm, suggesting that the cobalt ions exist in the same oxidation state in those materials. The absorption spectrum belonging Co_xO_y/ZSM-5 (**Figure S3F**) has a somewhat deviant shape, regarding the absorption band around 500 nm. For this reason, we do not refer to a specific oxidation state of Co (*i.e.* set x and y values in Co_xO_y) when discussing the Co_xO_y/ZSM-5 composite material. For all other composite materials, it is assumed that cobalt oxide is present as Co_3O_4.

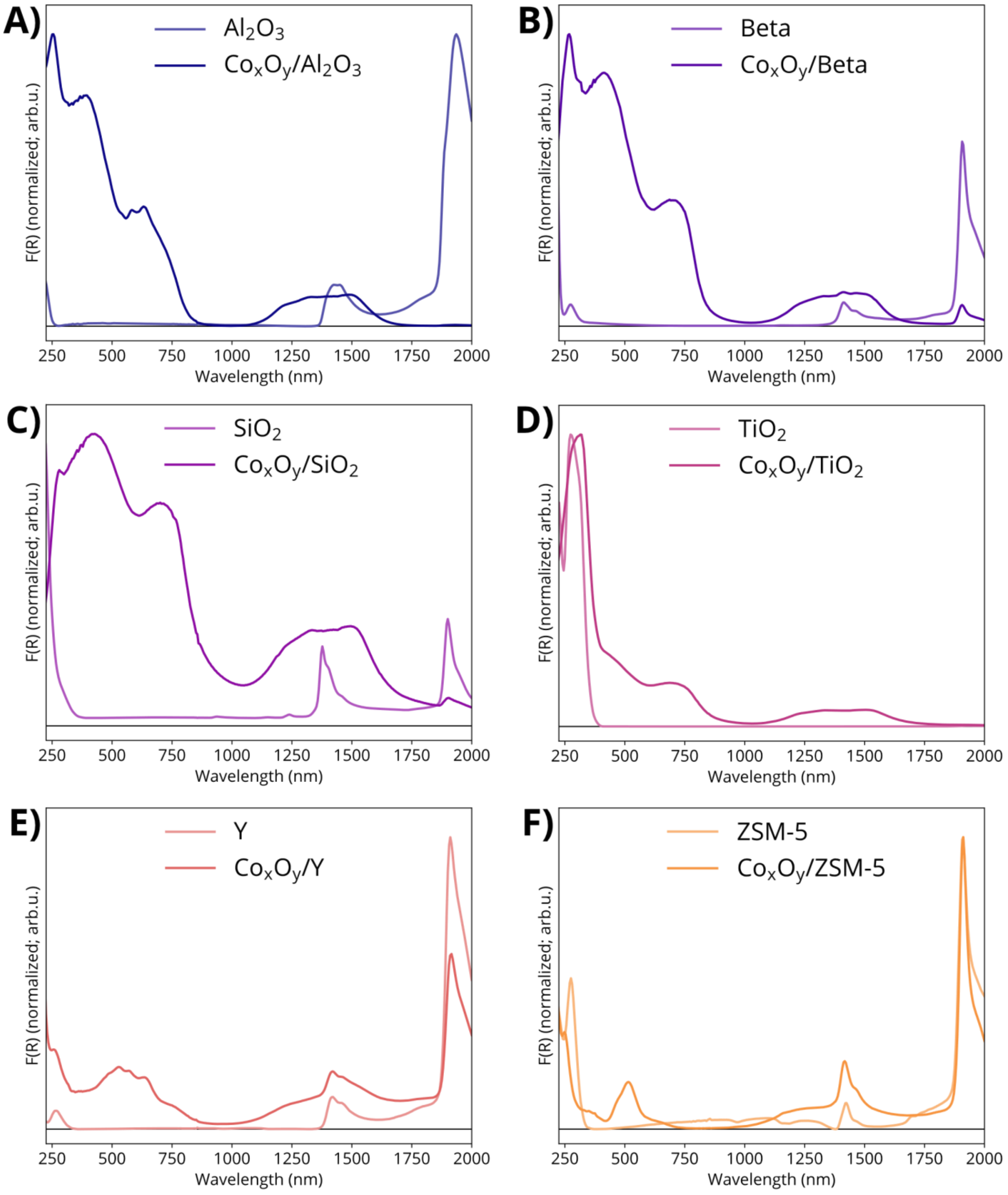

Figure S3. UV-Vis-NIR diffuse reflectance spectra of composite materials consisting of Co_xO_y and A) Al_2O_3; B) zeolite Beta; C) SiO_2; D) TiO_2; E) zeolite Y; F) zeolite H-ZSM-5.

Dye decolorization: selecting the best performing material

Figure S4 shows that composite materials of Co_xO_y with zeolite Beta, zeolite Y, zeolite ZSM-5 and SiO_2 do not decolorize the dye under solar light illumination or in the dark. Co_xO_y/Al_2O_3 appears decolorize the dye, but **Figure S4A** shows that this sample has similar activity in the dark. Therefore, this effect is not assumed to be light-related. Only Co_xO_y on TiO_2 shows a significant difference between light and dark conditions. Therefore, Co_xO_y/TiO_2 was determined to be the best performing material. Later, as shown in Figure 2 of the main text, it was determined that a mixed-phase cobalt(II,III) oxide (Co_3O_4) was present when forming a Co_xO_y/TiO_2 composite material.

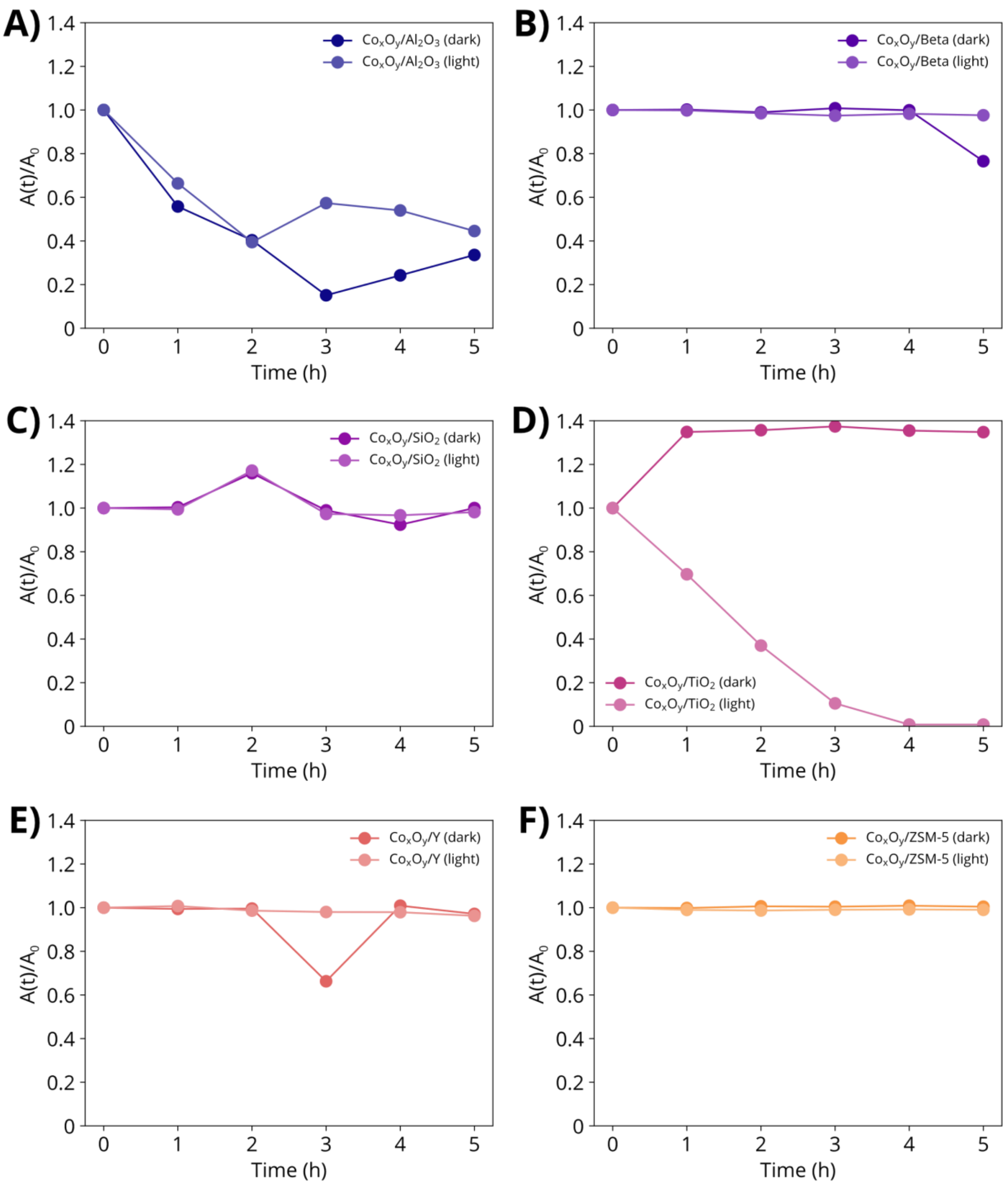

Figure S4. Indigo carmine decolorization experiments performed using composite materials consisting of Co_xO_y and A) Al_2O_3; B) zeolite Beta; C) SiO_2; D) TiO_2; E) zeolite Y; F) zeolite H-ZSM-5. The degree of dye decolorization is expressed as $A(t)/A_0$. For all materials, the experiment is shown under dark and light (simulated solar light illumination) conditions.

7. Dye decolorization: absolute absorption values

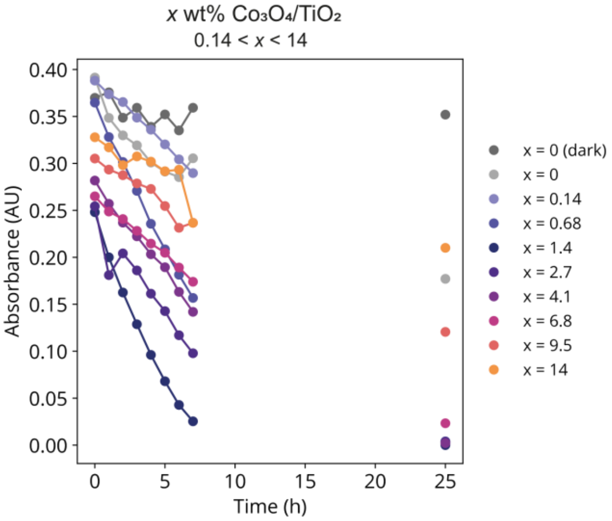

Figure S5. Absolute values of the indigo carmine dye absorbance data shown in main text Figure 3A and 3B. Indigo carmine dye is decolorized under green light illumination (λ_{max} = 520 nm) using x wt% Co_3O_4/TiO_2 photocatalyst materials (0.14 < x < 14). Bare TiO_2 (x = 0) is also shown under light and dark conditions. Between 7 h and 25 h, the dye concentration was not measured.

Figure S5 shows the absolute dye absorbance values of the data shown in Figures 3A and 3B of the main text, where the absorbance data is normalized to t = 0. Before performing the dye decolorization reaction, the indigo carmine dye solution and catalyst were mixed and stirred in the dark overnight. The absolute light absorbance values of the dye molecule before illumination differs between experiments using different catalysts, because a different equilibrium exists between dye molecules in solution and dye molecules adsorbed to the catalyst surface for different catalysts.

8. Effect of light on dye when there is no catalyst present

The dye was subjected to simulated solar light and green light in the absence of any catalyst to determine if the dye self-decolorizes under the influence of light only. **Figure S6** shows that the absorption spectra of the dye change only minimally after several hours of illumination under both used light sources.

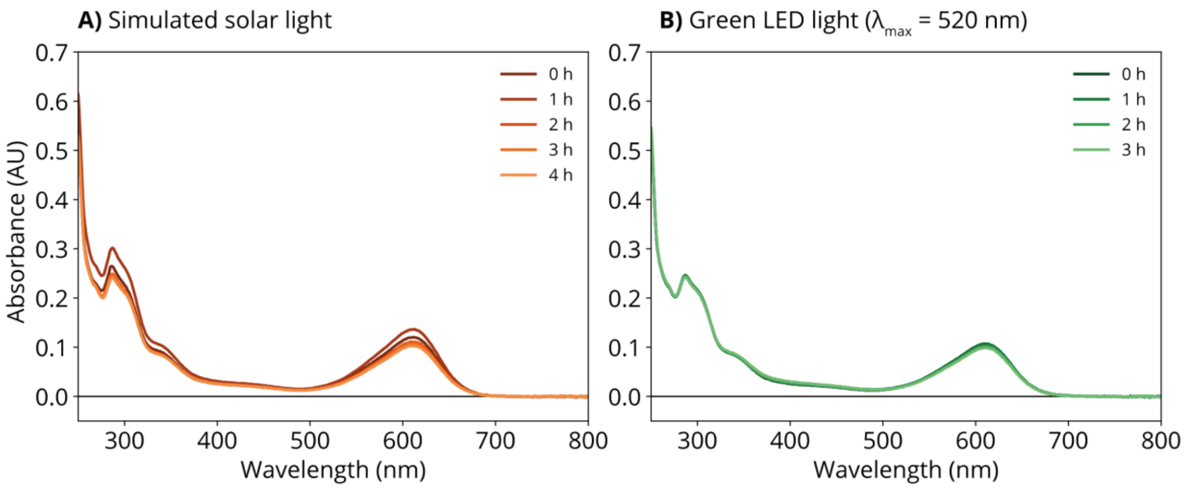

Figure S6. 100 ppm indigo carmine dye in water without catalyst under A) simulated solar light and B) green LED light conditions.

9. Dye decolorization: fitting data

An exponentially progressing dye decolorization reaction can be described via the following equation:

$$A(t)/A_0 = Ce^{-kt}, \qquad \text{(Eq. S1)}$$

where $A(t)/A_0$ is the absorbance relative to the starting absorbance A_0, C is the constant pre-exponential factor, t is the illumination time, and k is the apparent rate constant, which is related to the lifetime. Eq. S1 can be rewritten to the form:

$$\ln(A_0/A) = kt + b, \quad \text{(Eq. S2)}$$

where b is a constant equal to zero. Eq. S2 is in linear form, where the slope is k and the intercept is b. Therefore, the natural logarithm of the relative dye concentration was fit to a linear function as in Eq. S2.

Since there no data points were collected between t = 7 h and t = 25 h, photocatalyst materials that (almost) fully decolorized the dye overnight may have already completed the decolorization process between 7 h and 25 h. Including the t = 25 h data point would then give an inaccurate view of the dye decolorization process. Additionally, for samples where $A(t)/A_0 = 0$, $\ln(A_0/A(t))$ yields an infinitely high value. The t = 25 h data point was thus left out of the fitting procedures for all samples to ensure the best comparability between samples. The results are shown in **Figure S7**.

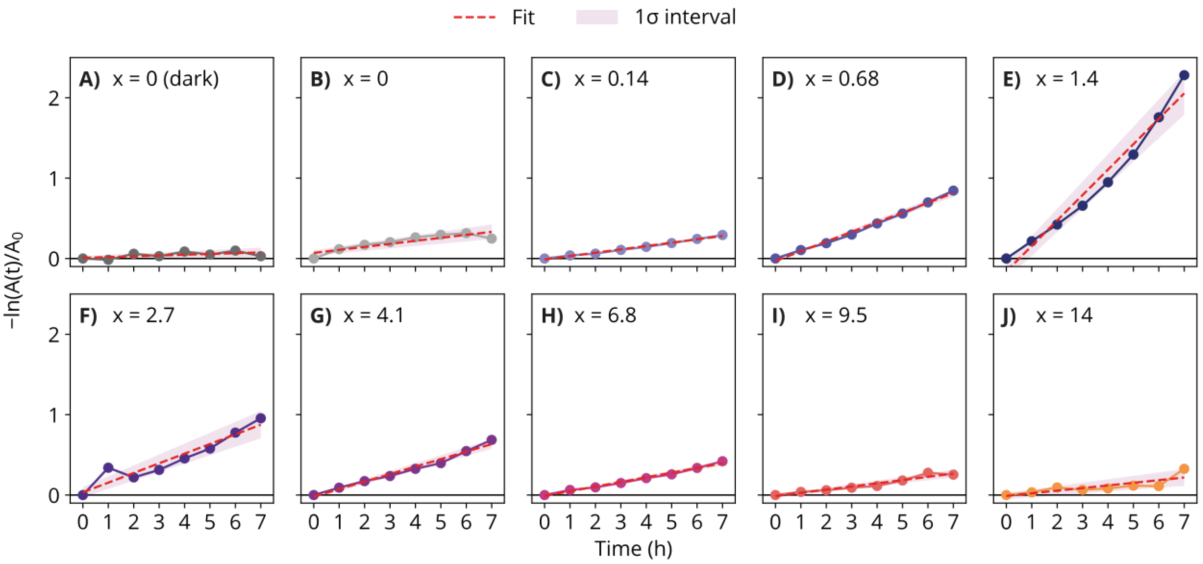

Figure S7. –ln(A(t)/A₀) as a function of illumination time, including a linear fit in the form of $-\ln(A(t)/A_0) = kt + b$, where $\ln(A(t)/A_0)$ is the natural logarithm of the normalized absorbance after decolorization experiments performed on x wt% Co_3O_4/TiO_2. The red dashed line indicates the fit and the area shaded in pink indicates one standard deviation (σ) around the fit values, as determined from the covariance matrix by taking the square root of the diagonal values.

10. Dye decolorization: solar versus green light

Figure S8 shows dye decolorization reactions performed on bare TiO_2, 1.4 wt% Co_3O_4/TiO_2 and 4.1 wt% Co_3O_4/TiO_2 under dark, simulated solar light and green LED light conditions. In the dark, TiO_2 is not photoactive. Under solar light conditions, both the 1.4 wt% and 4.1 wt% Co_3O_4/TiO_2 materials show a significantly higher rate of dye decolorization than the bare TiO_2. Under green light conditions however, the 4.1 wt% material shows a decolorization rate similar to that of bare TiO_2, while the 1.4 wt% material shows a significantly higher rate of dye decolorization.

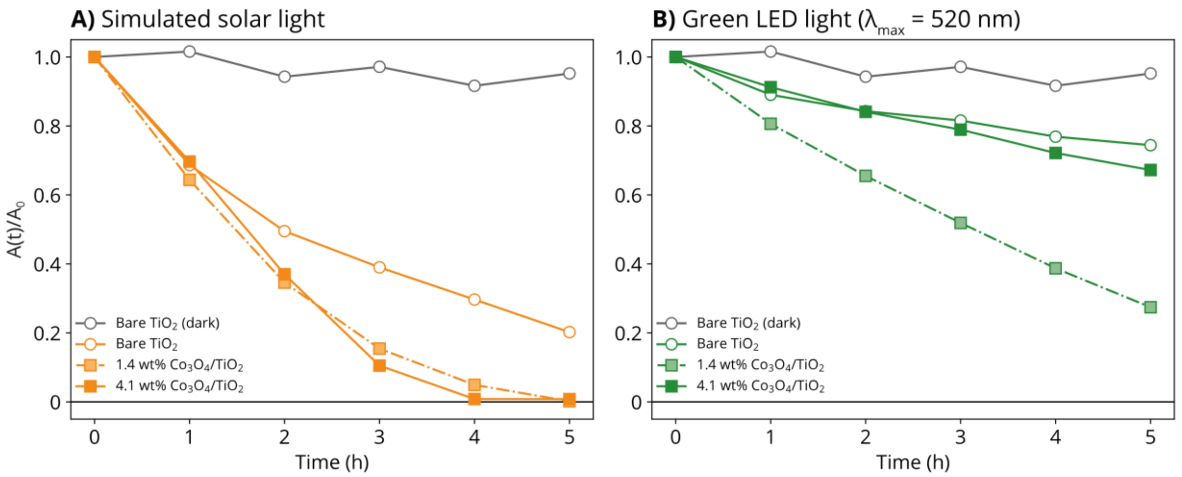

Figure S8. Dye decolorization experiments performed with bare TiO_2 (◯) and 1.4 or 4.1 wt% Co_3O_4/TiO_2 (□) under A) simulated solar light and B) green LED light conditions. The gray data indicates no light illumination.

11. X-ray diffraction measurements

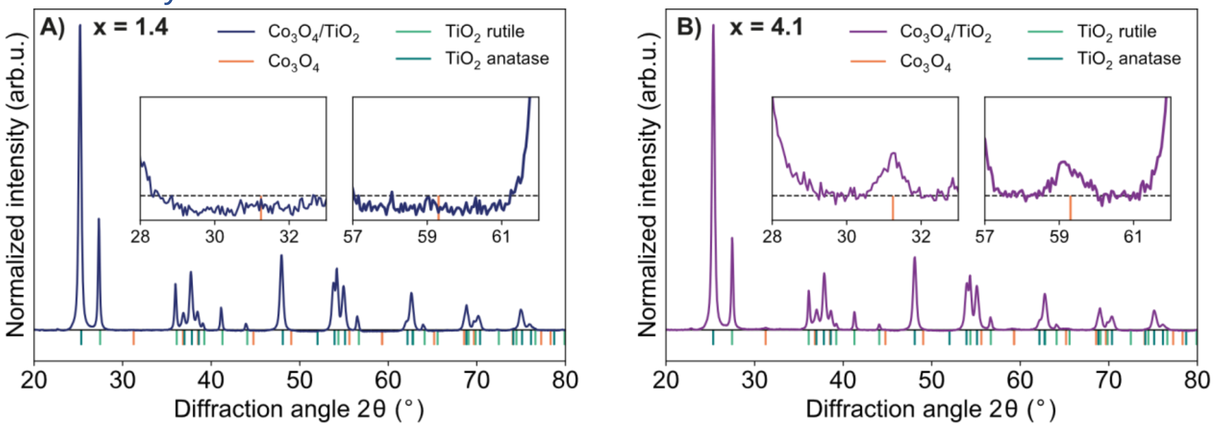

Figure S9. X-ray diffraction (XRD) patterns of x wt% Co_3O_4/TiO_2 (A: x = 1.4, B: x = 4.1). The XRD patterns were collected overnight to increase visibility of any Co_3O_4 reflections, if present. A) For x = 1.4, reflections characteristic of Co_3O_4 were not observed. However, TiO_2 reflections were observed across the whole measurement range, indicating that the structure of TiO_2 remained intact after the wet impregnation procedure. B) For x = 4.1, reflections characteristic of Co_3O_4 were observed around 2θ = 31° and 59°, as shown in the insets.

In **Figure S9A**, no reflections characteristic of Co_3O_4 were observed. This was the case for all x wt% Co_3O_4/TiO_2 materials with x < 4.1. However, TiO_2 reflections were observed across the whole measurement range, indicating that the structure of TiO_2 remained intact after the wet impregnation procedure. **Figure S9B** shows that for x = 4.1, reflections characteristic of Co_3O_4 were observed around 2θ = 31° and 59°, as shown in the insets. The Scherrer equation can be used to estimate the average crystallite size:

$$D = \frac{k\lambda}{\beta\cos(\theta)}, \qquad \text{(Eq. S3)}$$

where D is the average crystallite size, K is shape factor, typically set at 0.9, λ is the source wavelength (1.5418 Å), β is the full width half maximum of the chosen reflection (°), and θ is the diffraction angle (°). Here, the reflection at 2θ = 31° was chosen to calculate D, yielding an average crystallite size of 12 nm.

12. Transmission electron microscopy measurements

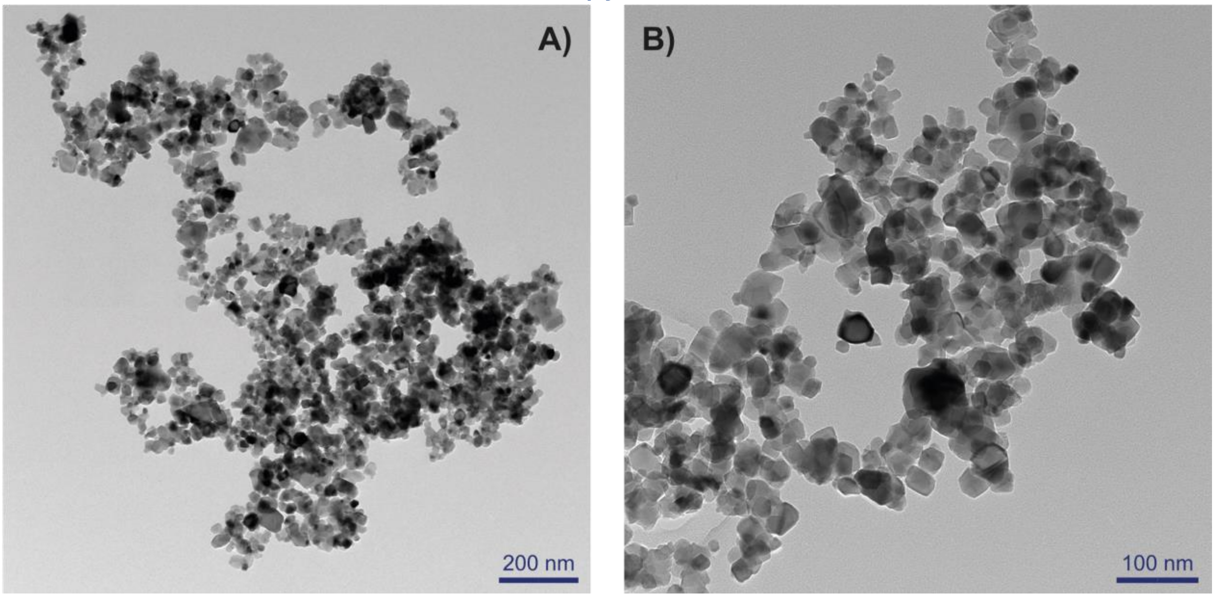

Figure S10. Transmission electron microscopy (TEM) image of a 4.1 wt% Co_3O_4/TiO_2 catalyst at two different magnifications.

Transmission electron microscopy (TEM) images were collected on a Tecnai TF-20 instrument at 200 kV (**Figure S10**). The average crystallite size is slightly bigger than was estimated from XRD. Since Co and Ti have similar electron scattering properties, it is difficult to discern Co from Ti in these TEM images.

13. Lamp emission spectra versus dye absorption spectrum

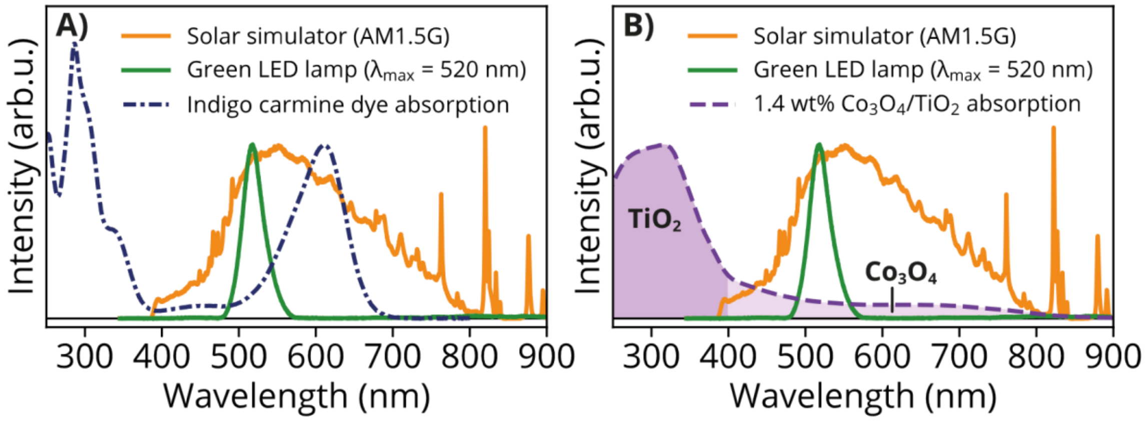

Figure S11. A) Emission spectra of the used light sources, a solar simulator with an AM1.5G filter (orange) and a green LED lamp (green) compared to the absorption spectrum of indigo carmine dye (blue). All spectra are normalized to the most intense emission or absorption value in the wavelength range of $400 < \lambda < 900$ nm. B) Emission spectra of the used solar simulator with an AM1.5G filter (orange) and green LED lamp (green) compared to the absorption spectrum of 1.4 wt% Co_3O_4/TiO_2 (purple; diffuse reflectance spectroscopy, corrected through the Schuster-Kubelka-Munk equation). All spectra are normalized to the most intense emission or absorption value in the wavelength range of $250 < \lambda < 900$ nm.

Figure S11A shows the emission spectra of the solar simulator and green LED lamp used for dye decolorization experiments, as well as the indigo carmine dye absorption spectrum. The solar simulator is an A1-LA125 LightLine Fiberized Solar Simulator (ScienceTech) equipped with an AM1.5G filter, used at 1 Sun intensity (100 mW/cm^2 at a power setpoint of 83.5%) at 14 cm working distance, *i.e.*, the distance between the liquid surface and the lamp surface was adjusted at 14 cm. The green LED (Eurolite, LED IP FL-30 SMD, 30 W, λ_{max} = 520 nm) was also used at a working distance of 14 cm and

the total absolute irradiance at this distance was measured to be 5.2 mW/cm^2. Due to detector limitations, wavelengths below 400 nm are not shown for the emission spectra. **Figure S11**A shows that there is overlap between both lamp emission spectra and the dye absorption spectrum, allowing both light sources to excite the dye molecule.

The absorption spectrum of 1.4 wt% Co_3O_4/TiO_2 is compared to both the solar simulator and the green LED lamp emission spectra in **Figure S11**B. Wavelengths in the UV-range (λ < 400 nm) are mainly absorbed by TiO_2 (dark purple shading), and TiO_2 does not absorb in the visible range of wavelengths (400 < λ < 700 nm). Since the green lamp only emits in the visible range, its emission does not overlap with the absorption of TiO_2. Co_3O_4, however, does absorb in the visible range (light purple shading): all photons emitted by the green lamp emission are absorbed by Co_3O_4. The green lamp therefore exclusively excites Co_3O_4 in the Co_3O_4/TiO_2 materials.

The solar simulator contains a small fraction of UV light, which is seen in **Figure S11**B as a small overlap between TiO_2 absorption and solar lamp emission. There is also a substantial overlap between the solar emission spectrum and the Co_3O_4 absorbance. Therefore, the solar simulator is able to excite both Co_3O_4 and TiO_2.

14. Dye degradation products detected by nuclear magnetic resonance

Proton nuclear magnetic resonance (^{1}H-NMR, further referred to as NMR) was used to aid in determining the cause of the dye decolorization. Dyes can be decolorized due to a structural change, *i.e.*, dye degradation (*i.e.*, partial or complete chemical decomposition), but dye decolorization can also be a result of, *e.g.*, an accepted electron, as is the case for methyl viologen dye.[22] By comparing NMR spectra before and during the dye decolorization process, it can be determined if the dye is structurally degraded or only decolorized because of an excess (or deficiency) of electrons. Figure 4A of the main text shows the structure of the indigo carmine dye. Protons resulting in a unique NMR signal are each indicated with a different color. Additionally, **Table S3** shows the location of the signals in DMSO. In our case, where (deuterated) water is the solvent, it is highly probable that proton **a**, attached to the nitrogen atom, is quickly exchanged with the solvent and therefore is not visible in the NMR spectra. Additionally, the NMR signal positions may be shifted when a different solvent is used (*i.e.*, D_2O instead of DMSO).

Table S3. ^{1}H-NMR signals for indigo carmine dissolved in DMSO (literature)[59] and in D_2O (experimental).

Proton	Multiplicity	δ_{DMSO} (ppm)	δ_{D2O} (ppm)
**a**	s	10.7	-
**b**	s	7.827	8.10
**c**	d	7.779	7.90
**d**	d	7.280	7.17

NMR was measured after performing a dye decolorization reaction in D_2O, as described in Section 0, using a 1.4 wt% Co_3O_4/TiO_2 catalyst as well as bare TiO_2, under green LED light illumination (λ_{max} = 520 nm). Aliquots were taken at t = 0, t = 1 h, t = 2 h and t = 24 h. These parameters were chosen based on **Figure S8**B, where, after 2 h of green light illumination, about 35% of the dye was decolorized for 1.4 wt% Co_3O_4/TiO_2 and 10% decolorized for TiO_2 (Figure 4). Note that the concentration of the dye at t = 0 is around 100 ppm. In the case of dye degradation, this concentration decreases.

Figure S12 shows the relative absorbance $A(t)/A_0$ corresponding to the UV-Vis data shown in the insets of Figure 4B-K, similar to relative absorbance data as shown in Figure 3, for example. After 2 h, about 35% of the dye was decolorized for the 1.4 wt% Co_3O_4/TiO_2 catalyst and 10% for the TiO_2 catalyst, and the dye was fully decolorized in both cases after 24 h.

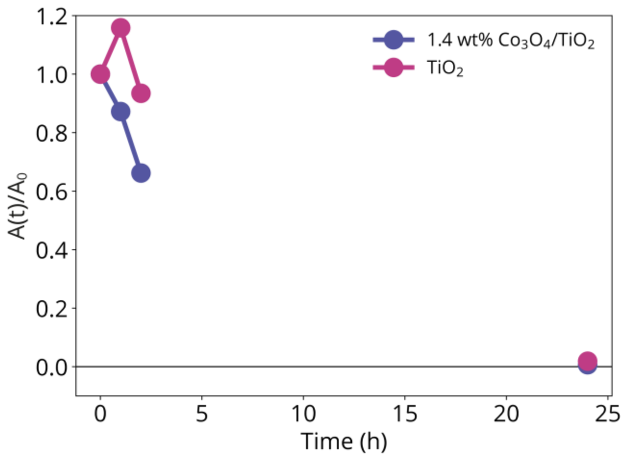

Figure S12. Relative absorbance $A(t)/A_0$ corresponding to the UV-Vis spectra shown in the insets of Figure 4B-K. No spectra were collected between 2 h and 24 h.

General observations on NMR data

The NMR data of Figure 4 is shown in greater detail in **Figure S13**A-E (1.4 wt% Co_3O_4/TiO_2) and **Figure S14**A-E (bare TiO_2), including NMR peak assignment. For all NMR spectra in both of these figures, the singlet of proton **b** (red, δ 8.10 ppm) is split twice into a double doublet: once by weak coupling to proton **c** (blue, δ 7.90 ppm) with a coupling constant of J = 1.9 Hz, and once by another group of unknown identity. Due to low signal-to-noise ratio, the small coupling constant (J = 0.6 Hz) is not expressed in most spectra. The doublet resulting from proton **c** is also split into a double doublet, the smaller coupling constant (J = 1.9 Hz) correlating to coupling with proton **b**. The signal of proton **d** has the same coupling constant as proton **c** (J = 8.6 Hz).

The signal of proton **b** (δ 8.10 ppm) seems to shift slightly to the left (higher chemical shift; less shielding) for both catalysts. The signals of protons **c** and **d** (δ 7.92, 7.21 ppm, respectively) both shift significantly to higher chemical shift values over time. The internal standard signal does not shift. A shift in chemical shift can have several causes, as discussed in the main text. In addition to shifting in position, the signals shaded in **Figure S13** and **Figure S14** show some broadening, possibly due to the presence of some catalyst particles that remained in the solution after separating the solid catalyst from the reaction mixture through centrifugation.

The three signals from protons **b-d** were integrated and normalized to the area of the internal standard. Subsequently, the area of each peak was normalized to the area of that peak at t = 0 h – similar to the UV-Vis data in **Figure S12** – so that the relative area of each peak is equal to 1at t = 0 h (**Figure S13**F and **Figure S14**F).

NMR spectra in the paramagnetic range (-100 to 200 ppm) were also inspected, however, no signals of, *e.g.*, cobalt ions were detected. This indicates that the metal ions are not leaching, even after being in contact with the dye solution for several hours under both dark and light conditions.

1.4 wt% Co_3O_4/TiO_2

For 1.4 wt% Co_3O_4/TiO_2, the peak areas of all three protons decrease in a very similar trend (**Figure S13**F), showing a decrease in area of about 35% after 2 h of reaction time. This decrease matches the decrease observed in UV-Vis absorbance (**Figure S12**). It should be noted that the area decrease of the proton **c**-signal is not represented accurately, as overlapping signals were observed. This is indicated by the multiplet at δ 7.99-7.90, most clearly seen in **Figure S13**D. Assumably, this multiplet can be divided in two overlapping signals: (1) δ 7.93 (dd, J = 2.2, 0.34 Hz, 1H); and (2) δ 7.95/7.92 (dd, J = 8.6, 1.9 Hz, 1H). The double doublet (2) with J = 8.6, 1.9 Hz indeed originates from proton **c**. However, the double doublet with J = 2.2, 0.34 Hz does not originate from (part of) the intact indigo carmine molecule, as these coupling constants are different from those observed for any signals in the fresh dye. Therefore, this signal is assumed to belong to a reaction product.

In addition to the signals of protons **b-d**, several new signals appear during the decolorization reaction, around δ 7.71, 7.60, 6.94 and 6.88. The signals around δ 7.71 and 6.94 are already observed after the equilibration process in the dark, as seen in **Figure S13**B, indicating that some reaction already takes place in the dark. However, after 2 h, these signals have become significantly larger. Additionally, the signals around δ 7.60 and 6.88 are only visible after 24 h of decolorization reaction under illumination, indicating that some reaction product has formed between 2 and 24 h. The coupling constant of J = 8.6 Hz is the same as that observed for protons **c** and **d**, indicating that these reaction products are structurally similar to the original dye molecule. Additionally, the signals in the range of proton **b** (red) and **c** (blue) remain visible after 24 h. As mentioned before, the signals in the range of proton **c** may contain overlapping signals of reaction products. Since the signal of proton **d** has disappeared completely after 24 h, we hypothesize that the signals observed in the range of protons **b** and **c** originate from reaction products that are structurally similar to the original dye molecule. The presence of any signal in these ranges however indicates that the indigo carmine dye molecule has not been degraded completely to CO_2.

The UV-Vis data (**Figure S12**, Figure 4 UV-Vis insets) show that the main absorbance band has completely disappeared. However, NMR suggests that some reactant and/or products are still present after 24 h, indicating that complete disappearance of UV-Vis light absorption does not equate full chemical decomposition in this case.

TiO_2

Similarly to 1.4 wt% Co_3O_4/TiO_2, the peak areas of all three protons decrease very similarly when TiO_2 is used as the catalyst (**Figure S14**F). Additionally, the trend of this decrease matches the decrease observed in UV-Vis absorbance (**Figure S12**). However, the peak areas of NMR seem to be slightly more decreased (by ~ 10-20%) than those of UV-Vis (by ~ 10%). Interestingly, both in the NMR and in the UV-Vis spectra, the y-axis intensity is increased at t = 1 h compared to t = 0 h, indicating a higher absorbance (UV-Vis) or larger amount of this compound (NMR) is present. For the NMR data, as discussed in the previous subsection concerning the dye decolorization reaction using 1.4 wt% Co_3O_4/TiO_2, it is possible that a reaction product results in signals overlapping with those of protons **b-d**, resulting in a larger relative area in NMR.

A newly appearing signal around δ 7.71 is observed at t = 1 h (dd, J = 8.9, 2.1 Hz), which disappears again after 2 h. This signal is at the same location as observed for 1.4 wt% Co_3O_4/TiO_2, and also has the same larger coupling constant of 8.9 Hz, indicating that the same reaction product is formed for both catalysts. However, for 1.4 wt% Co_3O_4/TiO_2, this signal remains visible after 24 h, whereas it has completely disappeared for TiO_2. The same is observed for the signal at δ 6.94. Interestingly, for TiO_2, the NMR spectrum at t = 24 h shows no signals other than that of the internal standard, indicating that the indigo carmine molecule and its reaction products have been completely degraded. This also matches the UV-Vis data, which show no absorbance after 24 h.

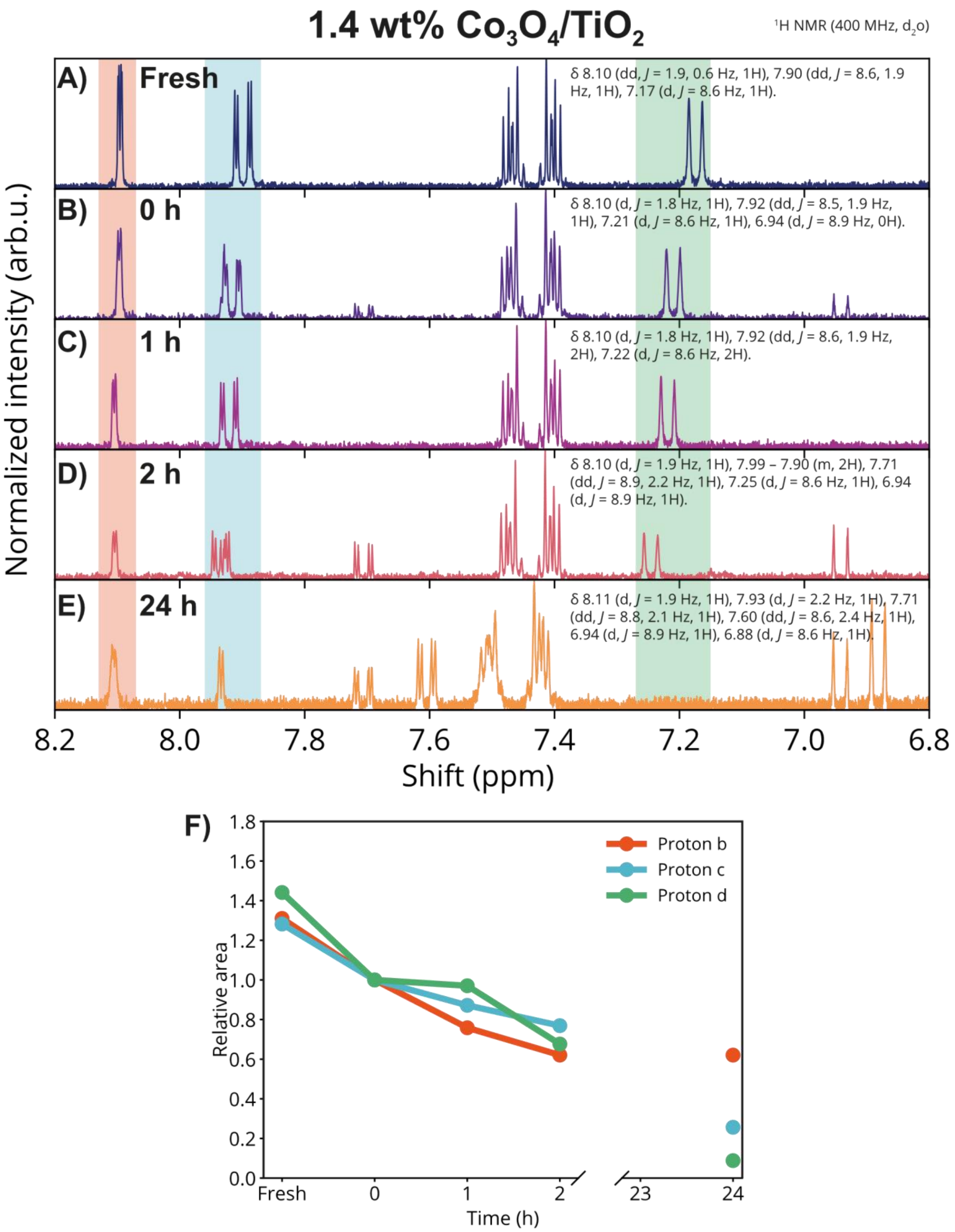

Figure S13. A-E) NMR spectra for the dye decolorization reaction performed using a 1.4 wt% Co_3O_4/TiO_2 catalyst under green light illumination, using D_2O as the solvent, including multiplet assignment. The internal standard was not included in the multiplet assignment. Signals originating from protons **b**, **c**, and **d** are indicated with red, blue, and green shading, respectively. A) fresh indigo carmine dye (100 ppm in D_2O), B) t = 0 h (directly before illumination), C) t = 1 h, D) t = 2 h, and E) t = 24 h after illumination with green LED light (λ_{max} = 520 nm). F) The relative peak area was determined by integrating each peak and normalizing to the area of the internal standard signal (δ 7.45 ppm), followed by normalizing to the peak area of the dye after equilibration in the dark (panel B).

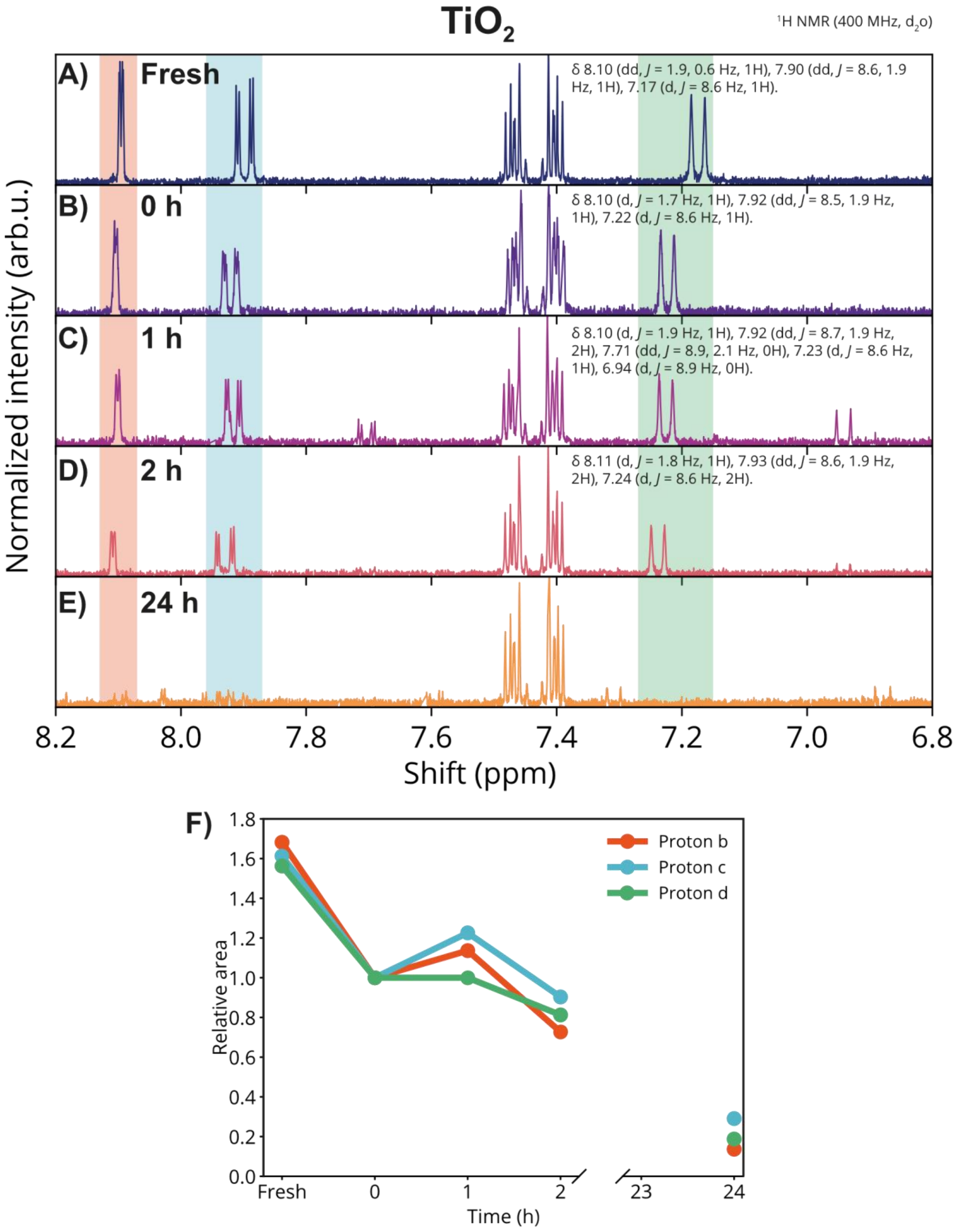

Figure S14. A-E) NMR spectra for the dye decolorization reaction performed using a bare TiO_2 catalyst under green light illumination, using D_2O as the solvent, including multiplet assignment. The internal standard was not included in the multiplet assignment. Signals originating from protons **b**, **c**, and **d** are indicated with red, blue, and green shading, respectively. A) fresh indigo carmine dye (100 ppm in D_2O), B) t = 0 h (directly before illumination), C) t = 1 h, D) t = 2 h, and E) t = 24 h after illumination with green LED light (λ_{max} = 520 nm). F) The relative peak area was determined by integrating each peak and normalizing to the area of the internal standard signal (δ 7.45 ppm), followed by normalizing to the peak area of the dye after equilibration in the dark (panel B).
